# Synthesis of Novel Carborane-Containing Derivatives of RGD Peptide

**DOI:** 10.3390/molecules28083467

**Published:** 2023-04-14

**Authors:** Alexander V. Vakhrushev, Dmitry A. Gruzdev, Alexander M. Demin, Galina L. Levit, Victor P. Krasnov

**Affiliations:** Postovsky Institute of Organic Synthesis, Russian Academy of Sciences (Ural Branch), 620108 Ekaterinburg, Russia; avv@ios.uran.ru (A.V.V.); amd2002@mail.ru (A.M.D.); ca512@ios.uran.ru (G.L.L.)

**Keywords:** 3-amino-1,2-dicarba-*closo*-dodecaborane, RGD peptide, linker fragment, protecting groups

## Abstract

Short peptides containing the Arg-Gly-Asp (RGD) fragment can selectively bind to integrins on the surface of tumor cells and are attractive transport molecules for the targeted delivery of therapeutic and diagnostic agents to tumors (for example, glioblastoma). We have demonstrated the possibility of obtaining the *N*- and *C*-protected RGD peptide containing 3-amino-*closo*-carborane and a glutaric acid residue as a linker fragment. The resulting carboranyl derivatives of the protected RGD peptide are of interest as starting compounds in the synthesis of unprotected or selectively protected peptides, as well as building blocks for preparation of boron-containing derivatives of the RGD peptide of a more complex structure.

## 1. Introduction

The search for efficient pharmaceuticals for the diagnostics and treatment of tumor diseases is one of the most urgent problems of medicinal chemistry. Currently, molecular vectors—namely, short peptides, antibodies, aptamers, and other compounds that provide targeted delivery of the functional part of the molecule—are widely used in the constructs of targeted therapy agents. The mechanism of their selective accumulation is based on the interaction of the vector with a target molecule, typically a receptor protein located on the surface of tumor cells.

**Figure 1:**
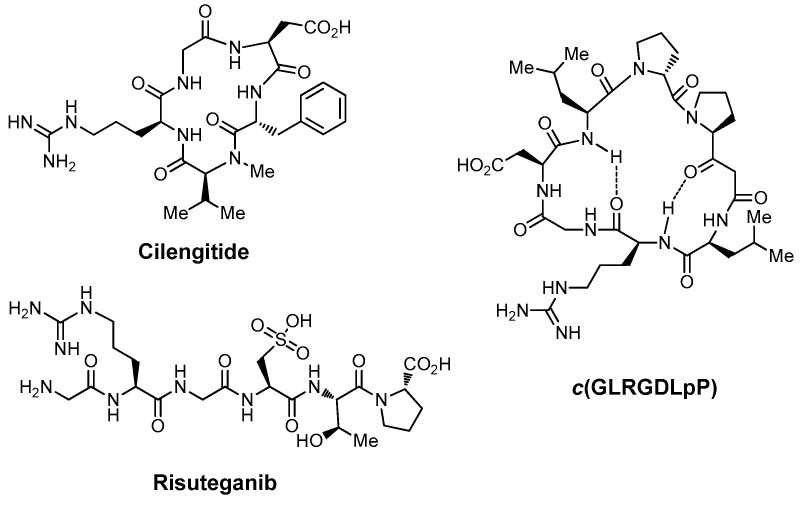
Biologically active compounds based on the RGD motif.

Today, the RGD peptide (l-arginyl-glycyl-l-aspartic acid, Arg-Gly-Asp) and structurally similar peptides (Figure 1) are widely used as molecular vectors in the drug design of targeted agents for the diagnostics and therapy of tumor diseases [1,2,3,4,5,6]. The RGD amino acid sequence has a tropism for cell adhesion proteins, integrins, which are particularly overexpressed in tumor cells (namely, αvβ_3_ and αvβ_5_ integrins). Integrin inhibitors represent an important class of agents for the treatment of tumors, macular degeneration, acute coronary syndrome, and other diseases [7,8]. Among the derivatives and analogs of the RGD peptide, a number of integrin inhibitors have been found [9,10]. Cilengitide, a selective inhibitor of αvβ_3_ and αvβ_5_ integrins proposed for the treatment of recurrent glioblastoma [11,12], has not passed phase III clinical trials because of insufficient pharmacokinetic parameters [13]. At the same time, studies of a number of other integrin inhibitors related to the RGD peptide are currently ongoing [14,15,16,17].

Based on the RGD peptide, a wide range of conjugates containing isotopic [18,19,20,21,22], fluorescent [23,24,25,26], or magnetic contrast labels [27,28,29], residues of cytostatic molecules [30,31,32,33,34], as well as agents for photodynamic therapy [35,36,37,38] have been synthesized. For efficient binding of the RGD peptide-based compounds to integrins (for example, on the surface of tumor cells), it is preferrable that the guanidine fragment of arginine and the carboxyl group of aspartic acid remain unsubstituted [39].

One of the emerging approaches to tumor treatment is boron neutron capture therapy (BNCT). This method is based on the ability of the ^10^B isotope to interact with thermal neutrons with the emission of ^4^He and ^7^Li nuclei, which locally damage cells containing boron compounds [40,41,42]. A crucial condition for the application of BNCT is the selective accumulation of boron-containing molecules by tumor cells. The design of low-toxic boron-containing tumor-targeting compounds is an urgent task of modern medicinal chemistry [43,44,45,46]. An important group of potential boron delivery agents are derivatives of 1,2-dicarba-*closo*-dodecaborane (carborane), the molecule of which contains ten boron atoms and can be modified using various functional groups. Certain properties of carboranes such as stability under physiological conditions and low toxicity make them unique pharmacophores for the design of new biomimetics [47,48,49]. Carborane conjugates with natural amino acids and peptides are of particular interest from the point of view of drug design of BNCT agents, as well as theranostic agents [50]. In particular, carborane-containing derivatives of the c(RGDfK) peptide have been used for adhesion of cells expressing the αvβ_3_ integrin receptors [51], as well as for boron delivery to tumor cells [52,53]. The boron-containing conjugate of the cyclic RGD peptide was able to selectively accumulate in murine SCCVII carcinoma cells but was highly toxic [53]. Boron-containing nanoparticles containing FITC-labeled RGD-K peptide residues [54] or internalizing RGD fragments [55,56] were selectively accumulated by ALTC1S1 glioma, GL261 glioma, and A549 adenocarcinoma cells. Modification of the sodium dodecaborate-loaded liposomes by c(RGDfK) [57,58] and c(RGDyC) [59] peptides made it possible to achieve their binding to human umbilical cord endothelial cells. The fact that RGD-functionalized *closo*-dodecaborate albumin conjugates are capable of accumulating in U87 MG xenografts has recently demonstrated the efficacy of BNCT in in vivo experiments [60]. The c(RGDfK) peptide-based theranostic agent containing both a dodecaborane residue and ^67^Ga and ^125^I isotope labels was highly stable and capable of accumulating in U87 MG glioblastoma cells [61].

Recently, we have demonstrated the possibility of obtaining carborane-containing derivatives and analogs of natural amino acids as a result of modifications of protected amino acids using classical methods of peptide chemistry (formation of an amide bond, selective introduction and removal of *N*- and *C*-protecting groups) [62,63,64,65,66,67].

**Scheme 1:**
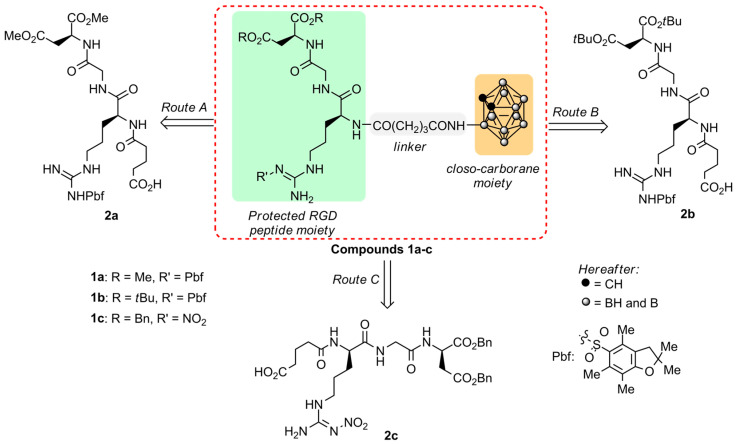
Synthetic routes to protected *closo*-carboranyl RGD peptide derivatives **1a**–**c**.

The purpose of this work was to synthesize new *N*- and *C*-protected derivatives of the RGD peptide containing a *closo*-carborane residue linked to the arginine α-amino group via a short linker (compounds **1a**–**c**, Scheme 1). We used a glutaric acid residue as a linker, which makes it possible to obtain conjugates of the RGD peptide with readily available 3-amino-*ortho*-carborane with a high boron content. The choice of protecting groups was due to the possibility of either selective deblocking of the guanidino group in the arginine residue and carboxyl groups in the aspartate residue (compound **2a**), or removal of all protecting groups in one step (compounds **2b,c**).

## 2. Results and Discussion

We have carried out a comparative study of three synthetic routes for *closo*-carboranyl derivatives of the RGD peptide involving the use of different protecting groups.

The synthesis of peptides **1a,b** was carried out starting from dimethyl and di-*tert*-butyl esters **2a** and **2b**, which we had previously obtained, containing a 2,2,4,6,7-pentamethyldihydrobenzofuran-5-sulfonyl (Pbf) group in the arginine side chain and a glutaryl fragment at the arginine α-amino group [68,69,70]. The protecting groups of compounds **1a** and **2a** can be removed selectively: ester groups by alkaline hydrolysis; and the Pbf group by the action of an acid, for example, TFA. Removal of the three protecting groups in compounds **1b** and **2b** can be carried out in one step, by acid treatment.

To obtain conjugate **1c**, it was necessary to synthesize a glutaryl derivative **2c** of the protected RGD peptide containing a nitro group in the guanidine fragment and two benzyl ester groups, which can be simultaneously removed by hydrogenolysis. The synthesis of derivatives of the RGD peptide containing benzyl aspartate and a nitro group protecting the side chain of arginine has been described in the literature; however, information on the physicochemical characteristics of intermediate compounds is fragmentary [71,72,73,74,75,76].

**Scheme 2:**
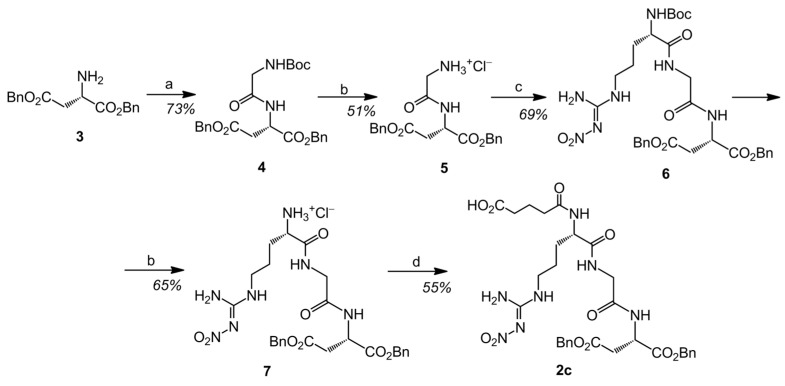
Synthesis of compound **2c**. (a) *N*-Boc-Gly-OH, HOSu, DIPEA, DCC, CH_2_Cl_2_, rt, 24 h; (b) HCl conc., MeOH, rt, 15 min; (c) *N*-Boc-Arg(NO_2_)-OH, DIPEA, TBTU, CH_2_Cl_2_, rt, 20 h; (d) glutaric anhydride, DIPEA, CH_2_Cl_2_, rt, 20 h.

We synthesized glutaryl derivative **2c** starting from dibenzyl (*S*)-aspartate (**3**) (Scheme 2). Coupling of amino ester **3** to *N*-Boc-glycine using *N*,*N*′-dicyclohexylcarbodiimide (DCC) as a coupling agent in the presence of *N*-hydroxysuccinimide (HOSu) and subsequent treatment of protected dipeptide **4** with hydrochloric acid in methanol led to amino ester **5** in moderate yield after chromatographic purification. Coupling of compound **5** to *N*^α^-Boc-*N*^ω^-nitro-(*S*)-arginine in the presence of TBTU gave the protected tripeptide **6**. Removal of the Boc group of compound **6** under acidic conditions and subsequent treatment of tripeptide **7** with glutaric anhydride gave compound **2c** containing a free carboxyl group.

At each stage of the synthesis of glutaryl tripeptide **2c**, the formation of side products was observed, so in order to obtain pure compounds **2c**, **4**–**7**, it was necessary to perform chromatographic purification. It is known that peptides containing an aspartic acid residue, including those in the RGD fragment, are prone to degradation, isomerization, and epimerization [77,78,79,80,81]. In our case, the total yield of compound **2c** (Scheme 2) was only 9.2% relative to the starting amino ester **3**. At the same time, the total yields of peptides **2a** and **2b** obtained from dimethyl and di-*tert*-butyl (*S*)-aspartates were about 20% [69].

**Scheme 3:**
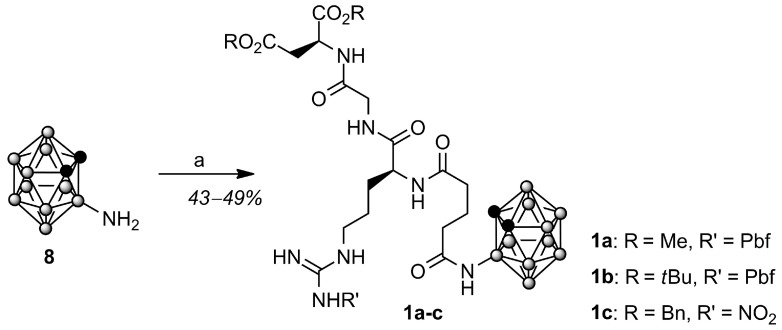
Synthesis of protected RGD peptide conjugates **1a**–**c**. (a) **2a**–**c**, EtOCOCl, NMM, CH_2_Cl_2_, −5 °C to rt, 16 h.

Coupling of compounds **2a**–**c** to 3-amino-*ortho*-carborane (**8**) by the mixed anhydride method in the presence of ethyl chloroformate led to protected carboranyl peptides **1a**–**c** in moderate yields (Scheme 3). Attempts to implement an alternative approach consisting in the acylation of amine **8** with glutaric anhydride followed by coupling to peptide **7** failed because of the low nucleophilicity of 3-aminocarborane.

Conjugates **1a**–**c** are colorless crystalline compounds that are stable during storage. Their ^1^H NMR spectra contain characteristic signals of the 3-aminocarborane protons: singlets at δ 8.21–8.25 ppm (amino group) and δ 5.05–5.06 ppm (two CH groups in the cluster) as well as wide multiplets at δ 1.1–2.6 ppm (9 BH groups). The ratio of the integral intensities of the signals of boron atoms in the ^11^B NMR spectra of peptides **1a**–**c** is 4:1:2:3 and corresponds to the symmetrical structure of 3-substituted *closo*-carborane.

To remove protecting groups in compounds **1a**–**c**, rather mild conditions are usually suitable, in which, as a rule, cleavage of peptide bonds or degradation of the *closo*-carborane residue do not occur. Thus, these derivatives can be considered as convenient starting compounds for further modifications.

## 3. Conclusions

Thus, we synthesized several protected derivatives of the RGD peptide containing 3-amino-*closo*-carborane and glutaryl residue as a linker. The structural motif of the RGD peptide can be considered as a basis for the synthesis of potential boron delivery agents for BNCT; at the same time, the preparation of compounds of this group requires careful selection of reaction conditions. The derivatives obtained by us differ in the structure of the protecting groups; their removal can be carried out both in one stage (by hydrogenolysis or acidic treatment) and separately. This opens up prospects for further modification of the peptide fragment and the synthesis of carborane-containing peptides of a more complex structure.

## 4. Materials and Methods

Dimethyl (*S*,*S*)-(*N*^α^-4-carboxybutanoyl-*N*^ω^-Pbf-arginyl)-glycyl-aspartate (**2a**) [69], di-*tert*-butyl (*S*,*S*)-(*N*^α^-4-carboxybutanoyl-*N*^ω^-Pbf-arginyl)-glycyl-aspartate (**2b**) [69], dibenzyl (*S*)-aspartate 4-toluenesulfonate (**3**) [82], and 3-amino-1,2-dicarba-*closo*-dodecaborane (**8**) [83] were obtained according to known procedures. Other reagents were commercially available and were purchased from Alfa Aesar (Heysham, UK). Solvents were purified according to traditional methods [84] and used freshly distilled. Melting points were obtained on a SMP3 apparatus (Barloworld Scientific, Staffordshire, UK). Optical rotations were measured on a Perkin Elmer M341 polarimeter (Perkin Elmer, Waltham, MA, USA). The ^1^H, ^11^B, and ^13^C NMR spectra were recorded on a Bruker Avance 500 instrument (Bruker, Karlsruhe, Germany) with operating frequencies of 500, 160, and 126 MHz, respectively, at ambient temperature using TMS as an internal standard and BF_3_·Et_2_O as an external standard. The NMR spectra of the compounds were obtained; see the Supplementary Materials, Figures S1–S19. CHN-Elemental analysis was performed using a Perkin Elmer 2400 II analyzer (Perkin Elmer, Waltham, MA, USA). Analytical TLC was performed using Sorbfil plates (Imid, Krasnodar, Russia). Flash column chromatography was performed using Silica gel 60 (230–400 mesh) (Alfa Aesar, Heysham, UK). The high-resolution mass spectra were obtained using a Bruker maXis Impact HD mass spectrometer (Bruker, Karlsruhe, Germany), with electrospray ionization at atmospheric pressure in positive or negative mode, with direct sample inlet (4 L/min flow rate). Analytical reversed-phase HPLC was carried out with an Agilent 1100 instrument (Agilent Technologies, Santa Clara, CA, USA) using a Kromasil 100-5-C18 column (Nouryon, Göteborg, Sweden) thermostated at 35 °C, with detection at 230 nm (compounds **1b**, **1c**, **2c**, **4**, **6**, and **7**) or 254 nm (compound **1a**), and a 0.8 mL/min flow rate; the mobile phases are indicated in each specific case. For the HPLC data for compounds **4**, **6**, **7**, **2c**, and **1a**–**c**, see the Supplementary Materials, Figures S20–S26.

**Dibenzyl *N*-Boc-glycyl-(*S*)-aspartate (4).** DCC (0.48 g, 2.33 mmol) and DIPEA (1.22 mL, 6.98 mmol) were added to a solution of *N*-Boc-glycine (0.41 g, 2.33 mmol), dibenzyl (*S*)-aspartate 4-toluenesulfonate (**3**) (1.13 g, 2.33 mmol) and *N*-hydroxysuccinimide (0.13 g, 1.16 mmol) in CH_2_Cl_2_ (10 mL). The reaction mixture was stirred at room temperature for 24 h, and then filtered. The filtrate was successively washed with 10% citric acid solution (2 × 8 mL), saturated aqueous NaCl solution (2 × 8 mL), 5% aqueous NaHCO_3_ solution (2 × 8 mL), and saturated aqueous NaCl solution (8 mL). The organic layer was dried over Na_2_SO_4_ and evaporated to dryness under reduced pressure. The residue was purified by flash column chromatography (eluent benzene–EtOAc from 8:2 to 6:4). Yield, 0.79 g (73%). Colorless powder; m.p., 56 °C. [α]_D_^20^ + 9.9 (*c* 1.0, CHCl_3_). TLC (benzene–EtOAc 3:1): *R*_f_, 0.44. RP-HPLC (MeCN–H_2_O 1:1, 230 nm): τ, 4.7 min. ^1^H NMR (DMSO-*d*_6_) (*major conformer*) δ (ppm): 1.38 (s, 9H, *t*Bu), 2.80 (dd, *J* = 16.6, 6.8 Hz, H-3B Asp), 2.90 (dd, *J* = 16.6, 6.2 Hz, H-3A Asp), 3.55–3.57 (m, 2H, 2×H-2 Gly), 4.74–4.79 (m, H-2 Asp), 5.07 (s, 2H, Bn), 5.09 (s, 2H, Bn), 6.99 (t, *J* = 6.1 Hz, 1H, NH Gly), 7.31–7.37 (m, 10H, Ar), 8.35 (d, *J* = 8.0 Hz, 1H, NH Asp). ^13^C NMR (DMSO-*d*_6_) (*major conformer*) δ (ppm): 28.1 (3C), 35.8, 42.9, 48.5, 65.8, 66.2, 78.0, 127.6 (2C), 127.9 (2C), 128.0 (2C), 128.4 (2C), 128.4 (2C), 135.6, 135.7, 155.7, 169.4, 169.8, 170.3. Calcd (%) for C_25_H_30_N_2_O_7_: C, 63.82; H, 6.43; N, 5.95. Found (%): C, 63.89; H, 6.47; N, 5.99. HRMS (ESI) (*m*/*z*) [M+H]^+^: calcd for [C_25_H_31_N_2_O_7_]^+^: 471.2126; found: 471.2127.

**Dibenzyl *N*-Glycyl-(*S*)-aspartate Hydrochloride (5).** Concentrated HCl (2.0 mL, 24.0 mmol) was added to a solution of compound **4** (1.13 g, 2.4 mmol) in MeOH (10 mL). The reaction mixture was stirred at room temperature for 15 min, then evaporated to dryness under reduced pressure. The residue was purified by flash column chromatography on silica gel (eluent CHCl_3_–EtOH from 100:0 to 1:1). Yield, 0.34 g (51%). Yellowish oil. [α]_D_^20^ +5.9 (*c* 1.0, CHCl_3_). TLC (CHCl_3_–EtOH 3:1): *R*_f_, 0.69. ^1^H NMR (DMSO-*d*_6_) (*major conformer*) δ (ppm): 2.88 (dd, *J* = 16.8, 6.8 Hz, H-3B Asp), 2.93 (dd, *J* = 16.8, 5.7 Hz, H-3A Asp), 3.56–3.65 (m, 2H, H-2 Gly), 4.82–4.86 (m, H-2 Asp), 5.09 (s, 2H, Bn), 5.12 (s, 2H, Bn), 7.31–7.40 (m, 10H, Ar), 8.09 (s, 3H, NH_3_^+^), 9.00 (d, *J* = 7.9 Hz, 1H, NH Asp). ^13^C NMR (DMSO-*d*_6_) (*major conformer*) δ (ppm): 35.7, 43.7, 48.6, 66.0, 66.5, 127.8 (2C), 128.0 (2C), 128.1 (2C), 128.4 (4C), 135.6, 135.7, 166.4, 169.7, 170.0. HRMS (ESI) (*m*/*z*) [M+H]^+^: calcd for [C_20_H_23_N_2_O_5_]^+^: 371.1602; found: 371.1604.

**Dibenzyl (*S*,*S*)-(*N*^α^-Boc-*N*^ω^-nitroarginyl)-glycyl-aspartate (6).** TBTU (0.45 g, 1.41 mmol) and DIPEA (1.46 mL, 4.36 mmol) were added to a solution of amino ester hydrochloride **5** (0.57 g, 1.41 mmol) and *N*^α^-Boc-*N*^ω^-nitro-(*S*)-arginine (0.45 g, 1.41 mmol) in CH_2_Cl_2_ (20 mL). The reaction mixture was stirred at room temperature for 20 h then successively washed with 10% citric acid solution (2 × 15 mL), saturated aqueous NaCl solution (2 × 15 mL), 5% aqueous NaHCO_3_ solution (2 × 15 mL) and saturated aqueous NaCl solution (10 mL). The organic layer was dried over Na_2_SO_4_ and evaporated to dryness under reduced pressure. The residue was purified by flash column chromatography on silica gel (eluent CHCl_3_–EtOH from 10:0 to 8:2). Yield, 0.65 g (69%). Colorless powder; m.p., 112 °C (lit. m.p.: 98–99 °C [85], 99–102 °C [70]). [α]_D_^20^ +4.0 (*c* 1.0, CHCl_3_). TLC (CHCl_3_–EtOH 3:1): *R*_f_, 0.49. RP-HPLC (MeCN–H_2_O–AcOH 80:20:0.0025, 230 nm): τ, 4.2 min. ^1^H NMR (DMSO-*d*_6_) (*major conformer*) δ (ppm): 1.37 (s, 9H, *t*Bu), 1.43–1.59 (m, 3H, H-3B and 2×H-4 Arg), 1.59–1.71 (m, 1H, H-3A Arg), 2.79 (dd, *J* = 16.6, 6.8 Hz, H-3B Asp), 2.90 (dd, *J* = 16.3, 6.4 Hz, H-3A Asp), 3.07–3.17 (m, 2H, 2×H-5 Arg), 3.71 (dd, *J* = 16.8, 5.6 Hz, 1H, H-2A Gly), 3.76 (dd, *J* = 16.8, 5.7 Hz, 1H, H-2A Gly), 3.91–3.95 (m, 1H, H-2 Arg), 4.74–4.78 (m, H-2 Asp), 5.07 (s, 2H, Bn), 5.09 (s, 2H, Bn), 6.96 (d, *J* = 7.8 Hz, 1H, N^α^H Arg), 7.31–7.38 (m, 10H, Ar), 7.55–8.25 (br. s, 2H, 2×N^ω^H Arg), 8.07 (dd, *J* = 5.7, 5.6 Hz, 1H, NH Gly), 8.42 (d, *J* = 7.9 Hz, 1H, NH Asp), 8.44–8.54 (br. s, 1H, N^ω^H Arg). ^13^C NMR (DMSO-*d*_6_) δ (ppm): 24.6, 28.2 (3C), 29.1, 35.8, 40.0, 41.6, 48.6, 53.9, 65.9, 66.3, 78.2, 127.7 (2C), 127.9 (2C), 128.0 (2C), 128.4 (4C), 135.7, 135.8, 155.4, 159.3, 168.8, 169.7, 170.3, 172.2. Calcd (%) for C_31_H_41_N_7_O_10_: C, 55.43; H, 6.15; N, 14.60. Found (%): C, 55.07; H, 6.26; N, 14.77. HRMS (ESI) (*m*/*z*) [M+H]^+^: calcd for [C_31_H_42_N_7_O_10_]^+^: 672.2988; found: 672.2983.

**Dibenzyl (*S*,*S*)-(*N*^ω^-Nitroarginyl)-glycyl-aspartate Hydrochloride (7).** Concentrated HCl (0.50 mL, 5.95 mmol) was added to a solution of compound **6** (0.20 g, 0.30 mmol) in MeOH (5 mL). The reaction mixture was stirred at room temperature for 15 min, then evaporated to dryness under reduced pressure. The residue was purified by flash column chromatography on silica gel (eluent CHCl_3_–EtOH from 10:0 to 3:7). Yield, 0.12 g (65%). Yellowish powder; m.p., 61–64 °C. [α]_D_^20^ +17.6 (*c* 1.0, CHCl_3_). TLC (CHCl_3_–EtOH 3:1): *R*_f_, 0.28. RP-HPLC (MeCN–H_2_O–CF_3_CO_2_H 70:30:0.01, 230 nm): τ, 5.1 min. ^1^H NMR (DMSO-*d*_6_) δ (ppm): 1.47–1.61 (m, 2H, 2×H-4 Arg), 1.67–1.77 (m, 2H, 2×H-3 Arg), 2.82 (dd, *J* = 16.6, 7.0 Hz, H-3B Asp), 2.91 (dd, *J* = 16.6, 6.1 Hz, H-3A Asp), 3.18 (br. s, 2H, 2×H-5 Arg), 3.82–3.88 (m, 2H, 2×H-2 Gly and H-2 Arg), 4.76–4.80 (m, H-2 Asp), 5.08 (s, 2H, Bn), 5.10 (s, 2H, Bn), 7.31–7.38 (m, 10H, Ar), 7.68–8.23 (br. s, 2H, NH_2_Arg), 8.14 (s, 3H, NH_3_^+^), 8.47–8.63 (br. s, 1H, NH Arg), 8.65 (d, *J* = 7.9 Hz, 1H, NH Asp), 8.72 (t, *J* = 5.3 Hz, 1H, NH Gly). ^13^C NMR (DMSO-*d*_6_) δ (ppm): 24.4, 31.1, 35.8, 40.3, 41.5, 48.5, 53.6, 65.9, 66.3, 127.6 (2C), 127.9 (2C), 128.0 (2C), 128.4 (4C), 135.6, 135.7, 159.2, 168.8, 169.7, 170.3, 173.9. Calcd (%) for C_26_H_33_N_7_O_8_×1.5HCl: C, 49.86; H, 5.55; N, 15.66; Cl, 8.49. Found (%): C, 49.41; H, 5.54; N, 15.64; Cl, 8.24. HRMS (ESI) (*m*/*z*) [M+H]^+^: calcd for [C_26_H_34_N_7_O_8_]^+^: 572.2464; found: 572.2462.

**Dibenzyl (*S*,*S*)-(*N*^α^-Glutaryl-*N*^ω^-nitroarginyl)-glycyl-aspartate (2c).** A solution of compound **7** (0.30 g, 0.49 mmol), glutaric anhydride (0.056 g, 0.49 mmol) and DIPEA (0.13 mL, 0.74 mmol) in CH_2_Cl_2_ (5 mL) was stirred at room temperature for 20 h, then evaporated to dryness under reduced pressure. The residue was purified by flash column chromatography on silica gel (eluent CHCl_3_–EtOH from 9:1 to 3:7). Yield, 0.185 g (55%). Colorless powder; m.p., 103–108 °C. [α]_D_^20^ −1.7 (*c* 1.0, EtOH). TLC (CHCl_3_–EtOH 1:1): *R*_f_, 0.49. RP-HPLC (MeCN–H_2_O–AcOH 60:40:0.005, 230 nm): τ, 6.6 min. ^1^H NMR (DMSO-*d*_6_) δ (ppm): 1.41–1.58 (m, 3H, H-3B and 2×H-4 Arg), 1.62–1.74 (m, 3H, CH_2_ glutaryl and H-3A Arg), 2.14–2.20 (m, 4H, 2×CH_2_ glutaryl), 2.80 (dd, *J* = 16.6, 6.9 Hz, H-3B Asp), 2.90 (dd, *J* = 16.6, 6.3 Hz, H-3A Asp), 3.14 (br. s, 2H, 2×H-5 Arg), 3.71 (dd, *J* = 16.9, 5.7 Hz, 1H, H-2B Gly), 3.75 (dd, *J* = 16.9, 5.7 Hz, 1H, H-2A Gly), 4.19–4.23 (m, 1H, H-2 Arg), 4.74–4.78 (m, H-2 Asp), 5.07 (s, 2H, Bn), 5.09 (s, 2H, Bn), 7.31–7.38 (m, 10H, Ar), 8.16 (br. s, 2H, 2×N^ω^H Arg), 8.07 (d, *J* = 7.3 Hz, 1H, NH Asp), 8.24 (t, *J* = 5.7 Hz, 1H, NH Gly), 8.38 (d, *J* = 6.9 Hz, 1H, N^α^H Arg), 8.51 (br. s, 1H, N^ω^H Arg), 12.03 (s, 1H, CO_2_H). ^13^C NMR (DMSO-*d*_6_) δ (ppm): 20.6, 24.7, 28.9, 33.0, 34.2, 35.8, 40.2, 41.6, 48.5, 52.4, 65.9, 66.3, 127.7 (2C), 127.9 (2C), 128.0 (2C), 128.4 (4C), 135.7, 135.8, 159.3, 168.8, 169.7, 170.3, 172.0, 172.1, 174.2. Calcd (%) for C_31_H_39_N_7_O_11_: C, 54.30; H, 5.73; N, 14.30. Found (%): C, 53.94; H, 5.65; N, 13.99. HRMS (ESI) (*m*/*z*) [M−H]^−^: calcd for [C_31_H_38_N_7_O_11_]^−^: 684.2684; found 684.2685.

**General Procedure for the Synthesis of Carboranylaminoglutaryl Tripeptides 1a–c.** Ethyl chloroformate (63 μL, 0.66 mmol) was added to a cold (−10 °C) solution of an appropriate compound **2a**, **2b** or **2c** (0.66 mmol) and *N*-methylmorpholine (145 μL, 1.32 mmol) in CH_2_Cl_2_ (10 mL). The mixture was stirred at −10 °C for 15 min; then, 3-aminocarborane (**8**) (0.11 g, 0.66 mmol) was added. The reaction mixture was stirred at room temperature for 16 h, then successively washed with 10% citric acid solution (2 × 8 mL), saturated aqueous NaCl solution (2 × 8 mL), 5% aqueous NaHCO_3_ solution (2 × 8 mL) and saturated aqueous NaCl solution (8 mL). The organic layer was dried over Na_2_SO_4_ and evaporated to dryness under reduced pressure. The residue was purified by flash column chromatography (eluent CHCl_3_–EtOH from 10:0 to 8:2).

**Dimethyl (*S*,*S*)-{*N*^α^-[4-(1,2-Dicarba-*closo*-dodecaboran-3-yl)aminocarbonylbutanoyl]-*N*^ω^-Pbf-arginyl}-glycyl-aspartate (1a).** Yield, 0.28 g (48%). Colorless powder; m.p., 120–122 °C. [α]_D_^20^ +7.0 (*c* 0.9, CHCl_3_). TLC (CHCl_3_–EtOH 7:1): *R*_f_, 0.7. RP-HPLC (MeCN–H_2_O 1:1, 254 nm): τ, 8.2 min. ^1^H NMR (DMSO-*d*_6_) (*major conformer*) δ (ppm): 1.1–2.6 (br. m, 9H, 9×BH), 1.36–1.50 (m, 2H, 2×H-4 Arg), 1.41 (s, 6H, Pbf), 1.59–1.72 (m, 2H, CH_2_ glutaryl and 2×H-3 Arg), 2.01 (s, 3H, Pbf), 2.14 (t, *J* = 7.5 Hz, 2H, CH_2_ glutaryl), 2.19 (t, *J* = 7.4 Hz, 2H, CH_2_ glutaryl), 2.42 (s, 3H, Pbf), 2.48 (s, 3H, Pbf), 2.72 (dd, *J* = 16.6, 6.9 Hz, H-3B Asp), 2.80 (dd, *J* = 16.6, 6.2 Hz, H-3A Asp), 2.96 (s, 2H, Pbf), 3.00–3.04 (m, 2H, 2×H-5 Arg), 3.60 (s, 3H, CO_2_Me), 3.62 (s, 3H, CO_2_Me), 3.66–3.77 (m, 2H, 2×H-2 Gly), 4.17–4.21 (m, 1H, H-2 Arg), 4.65–4.69 (m, H-2 Asp), 5.06 (s, 2H, CH carborane), 6.37 (br. s, 1H, N^ω^H Arg), 6.56–7.12 (br. m, 2H, 2×N^ω^H Arg), 7.99 (d, *J* = 7.6 Hz, 1H, N^α^H Arg), 8.22 (t, *J* = 5.9 Hz, 1H, NH Gly), 8.23 (s, 1H, NH carborane), 8.28 (d, *J* = 7.8 Hz, 1H, NH Asp). ^11^B NMR (DMSO-*d*_6_) δ (ppm): −15.0 (br. s, 3B), −13.43 (2B), −10.69 (1B), −5.51 (4B). ^13^C NMR (DMSO-*d*_6_) (*major conformer*) δ (ppm): 12.2, 17.5, 18.8, 20.8, 25.4, 28.2 (2C), 29.0, 34.2, 35.6, 35.9, 41.5, 41.6, 42.4, 48.3, 51.6, 52.1, 57.1 (2C), 59.8, 86.2, 116.2, 124.2, 131.4, 134.1, 137.2, 156.0, 157.4, 168.6, 168.7, 170.2, 170.9, 172.0, 176.2. HRMS (ESI) (*m*/*z*) [M+H]^+^: calcd for [C_34_H_59_^11^B_10_N_7_O_11_S]^+^: 884.5044; found: 884.5045.

**Di-*tert*-butyl (*S*,*S*)-{*N*^α^-[4-(1,2-Dicarba-*closo*-dodecaboran-3-yl)aminocarbonylbutanoyl]-*N*^ω^-Pbf-arginyl}-glycyl-aspartate (1b).** Yield, 0.31 g (49%). Colorless powder; m.p., 126 °C. [α]_D_^20^ +5.5 (*c* 1.0, CHCl_3_). TLC (CHCl_3_–EtOH 7:1): *R*_f_, 0.71. RP-HPLC (MeCN–H_2_O–AcOH 40:60:0.0025, 230 nm): τ, 2.4 min. ^1^H NMR (DMSO-*d*_6_) (*major conformer*) δ (ppm): 1.1–2.6 (br. m, 9H, 9×BH), 1.32–1.51 (m, 2H, 2×H-4 Arg), 1.380 (s, 9H, *t*Bu), 1.384 (s, 9H, *t*Bu), 1.41 (s, 6H, Pbf), 1.58–1.66 (m, 1H, H-3B Arg), 1.66–1.76 (m, 3H, CH_2_ glutaryl and H-3A Arg), 2.01 (s, 3H, Pbf), 2.12–2.16 (m, 2H, CH_2_ glutaryl), 2.19 (t, *J* = 7.6 Hz, 2H, CH_2_ glutaryl), 2.42 (s, 3H, Pbf), 2.47 (s, 3H, Pbf), 2.54 (dd, *J* = 16.3, 6.9 Hz, H-3B Asp), 2.64 (dd, *J* = 16.3, 6.1 Hz, H-3A Asp), 2.96 (s, 2H, Pbf), 3.00–3.04 (m, 2H, 2×H-5 Arg), 3.68–3.74 (m, 2H, 2×H-2 Gly), 4.16–4.24 (m, 1H, H-2 Arg), 4.47–4.52 (m, H-2 Asp), 5.06 (s, 2H, CH carborane), 6.18–7.28 (m, 3H, 3×N^ω^H Arg), 7.98 (d, *J* = 7.5 Hz, 1H, N^α^H Arg), 8.13 (d, *J* = 8.0 Hz, 1H, NH Asp), 8.20 (t, *J* = 6.2 Hz, 1H, NH Gly), 8.23 (s, 1H, NH carborane). ^11^B NMR (DMSO-*d*_6_) δ (ppm): −15.0 (br. s, 3B), −13.46 (2B), −10.69 (1B), −5.52 (4B). ^13^C NMR (DMSO-*d*_6_) (*major conformer*) δ (ppm): 12.2, 17.5, 18.8, 20.8, 25.4, 27.5 (3C), 27.6 (3C), 28.2 (2C), 29.1, 34.2, 35.9, 37.1, 40.0 (overlapped by DMSO-*d*_6_ signal), 41.6, 42.4, 49.1, 52.3, 57.0 (2C), 80.4, 80.9, 86.2, 116.2, 124.2, 131.4, 134.1, 137.2, 156.0, 157.4, 168.5, 169.0, 169.5, 171.9, 172.0, 176.2. Calcd (%) for C_40_H_71_B_10_N_7_O_11_S: C, 49.72; H, 7.41; N, 10.15. Found (%): C, 49.65; H, 7.30; N, 9.98. HRMS (ESI) (*m*/*z*) [M+H]^+^: calcd for [C_40_H_72_^11^B_10_N_7_O_11_S]^+^: 968.5988; found: 968.5972.

**Dibenzyl (*S*,*S*)-{*N*^α^-[4-(1,2-Dicarba-*closo*-dodecaboran-3-yl)aminocarbonylbutanoyl]-*N*^ω^-nitroarginyl}-glycyl-aspartate (1c).** Yield, 0.23 g (43%). Colorless powder; m.p., 94–98 °C. [α]_D_^20^ +2.0 (*c* 1.0, CHCl_3_). TLC (CHCl_3_–EtOH 7:1): *R*_f_, 0.44. RP-HPLC (MeCN–0.06 M phosphate buffer solution (pH 7.0) 8:2, 230 nm): τ, 20.9 min. ^1^H NMR (DMSO-*d*_6_) (*major conformer*) δ (ppm): 1.2–2.6 (br. s, 9H, 9×BH), 1.42–1.57 (m, 3H, 2×H-4 and H-3B Arg), 1.62–1.75 (m, 3H, CH_2_ glutaryl and H-3A Arg), 2.13–2.19 (m, 4H, 2×CH_2_ glutaryl), 2.81 (dd, *J* = 16.6, 6.9 Hz, H-3B Asp), 2.90 (dd, *J* = 16.6, 6.3 Hz, H-3A Asp), 3.08–3.18 (m, 2H, 2×H-5 Arg), 3.68–3.78 (m, 2H, 2×H-2 Gly), 4.19–4.26 (m, 1H, H-2 Arg), 4.74–4.79 (m, H-2 Asp), 5.05 (s, 2H, 2×CH carborane), 5.07 (s, 2H, Bn), 5.09 (s, 2H, Bn), 7.31–7.38 (m, 10H, Ar), 7.52–8.30 (br. s, 2H, 2×N^ω^H Arg), 8.01 (d, *J* = 7.5 Hz, 1H, N^α^H Arg), 8.23 (m, 2H, NH carborane and NH Gly), 8.41 (d, *J* = 7.9 Hz, 1H, NH Asp), 8.51 (br. s, 1H, N^ω^H Arg). ^11^B NMR (DMSO-*d*_6_) δ (ppm): −15.0 (br. s, 3B), −13.42 (2B), −10.67 (1B), −5.51 (4B). ^13^C NMR (DMSO-*d*_6_) (*major conformer*) δ (ppm): 20.9, 24.7 (br. s), 29.0, 34.2, 35.7, 35.9, 40.2, 41.5, 48.5, 52.2, 57.1 (2C), 65.8, 66.3, 127.6 (2C), 127.8 (2C), 127.9, 128.0, 128.3 (4C), 135.6, 135.8, 159.2, 168.8, 169.6, 170.2, 171.9, 172.1, 176.2. HRMS (ESI) (*m*/*z*) [M+H]^+^: calcd for [C_33_H_51_^11^B_10_N_8_O_10_]^+^: 829.4699; found: 829.4694.

## Data Availability

Not applicable.

## Supplementary Materials

The following supporting information can be downloaded at: https://www.mdpi.com/article/10.3390/molecules28083467/s1, Figures S1–S19: ^1^H, ^11^B, and ^13^C NMR spectra of compounds **4–7**, **2c**, and **1a**–**c**; Figures S20–S26: HPLC data for compounds **4**, **6**, **7**, **2c**, and **1a**–**c**.
