# Supplementary material for: Synthesis of Novel Carborane-Containing Derivatives of RGD Peptide
Source: Molecules. 2023 Apr 14;28(8):3467. doi: 10.3390/molecules28083467 (PMC10143838; doi:10.3390/molecules28083467)
Supplement: Supplementary file 1 [file molecules-28-03467-s001.zip › molecules-2324439-supplementary.pdf]

Supplementary Materials

Synthesis of Novel Carborane-containing Derivatives of RGD Peptide

Alexander V. Vakhrushev [1], Dmitry A. Gruzdev [1,*], Alexander M. Demin [1], Galina L. Levit [1], and Victor P. Krasnov [1,*]

[1] Postovsky Institute of Organic Synthesis, Russian Academy of Sciences (Ural Branch), Ekaterinburg 620108, Russia

Table of Contents Page

NMR Spectra S2
Figure S1. ^{1}H NMR spectrum of compound **4** S2
Figure S2. ^{13}C NMR spectrum of compound **4** S2
Figure S3. ^{1}H NMR spectrum of compound **5** S3
Figure S4. ^{13}C NMR spectrum of compound **5** S3
Figure S5. ^{1}H NMR spectrum of compound **6** S4
Figure S6. ^{13}C NMR spectrum of compound **6** S4
Figure S7. ^{1}H NMR spectrum of compound **7** S5
Figure S8. ^{13}C NMR spectrum of compound **7** S5
Figure S9. ^{1}H NMR spectrum of compound **2c** S6
Figure S10. ^{13}C NMR spectrum of compound **2c** S6
Figure S11. ^{1}H NMR spectrum of compound **1a** S7
Figure S12. ^{11}B NMR spectrum of compound **1a** S7
Figure S13. ^{13}C NMR spectrum of compound **1a** S8
Figure S14. ^{1}H NMR spectrum of compound **1b** S8
Figure S15. ^{11}B NMR spectrum of compound **1b** S9
Figure S16. ^{13}C NMR spectrum of compound **1b** S9
Figure S17. ^{1}H NMR spectrum of compound **1c** S10
Figure S18. ^{11}B NMR spectrum of compound **1c** S10
Figure S19. ^{13}C NMR spectrum of compound **1c** S11

HPLC Data S12
Figure S20. HPLC of compound **4** S12
Figure S21. HPLC of compound **6** S12
Figure S22. HPLC of compound **7** S13
Figure S23. HPLC of compound **2c** S13
Figure S24. HPLC of compound **1a** S14
Figure S25. HPLC of compound **1b** S14
Figure S26. HPLC of compound **1c** S15

NMR Spectra

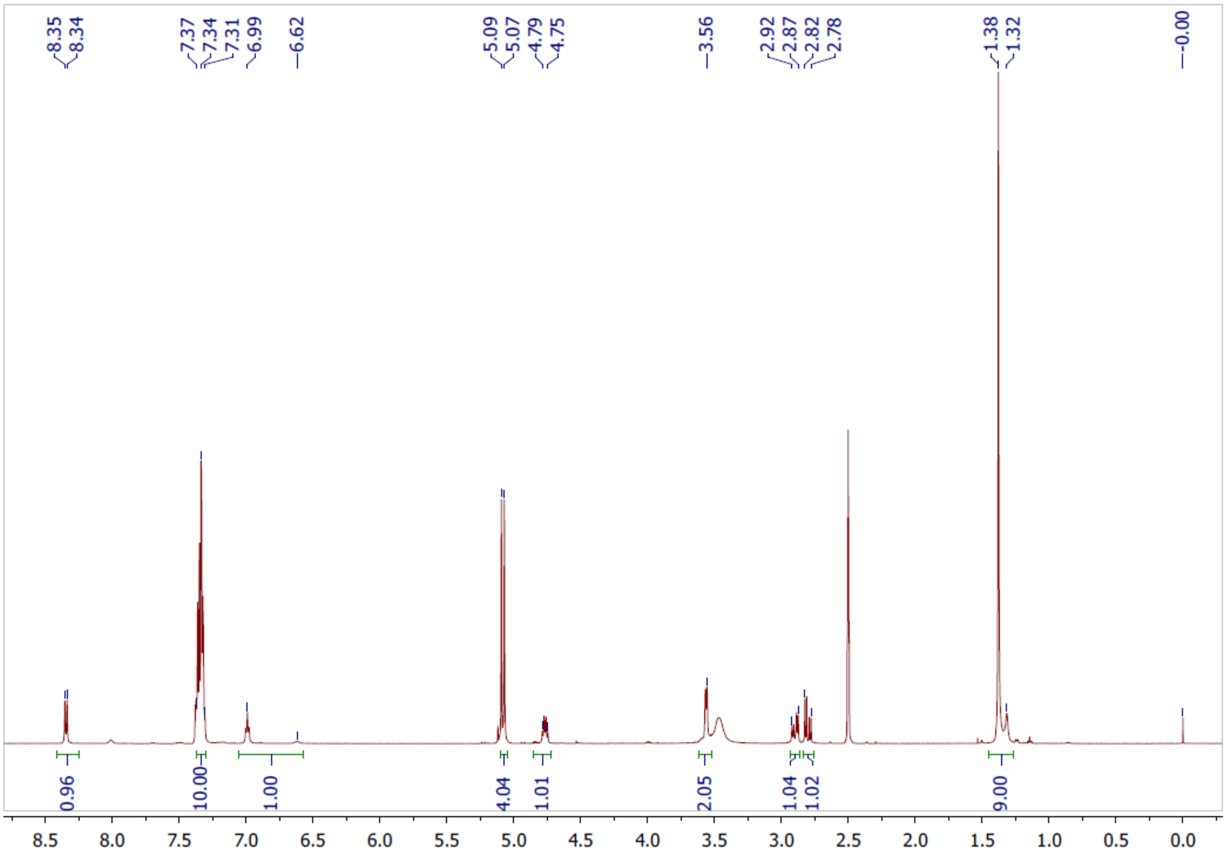

Figure S1. ^{1}H NMR spectrum of compound **4**

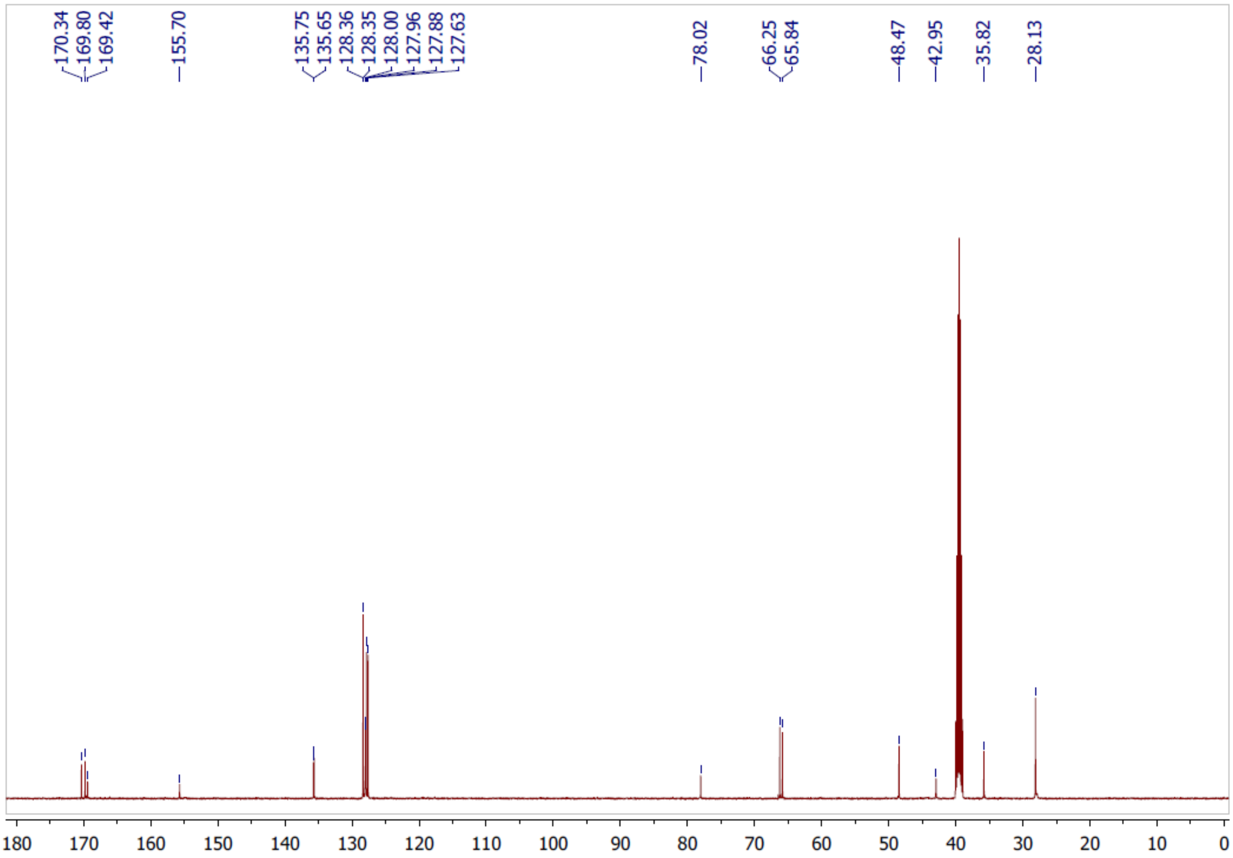

Figure S2. ^{13}C NMR spectrum of compound **4**

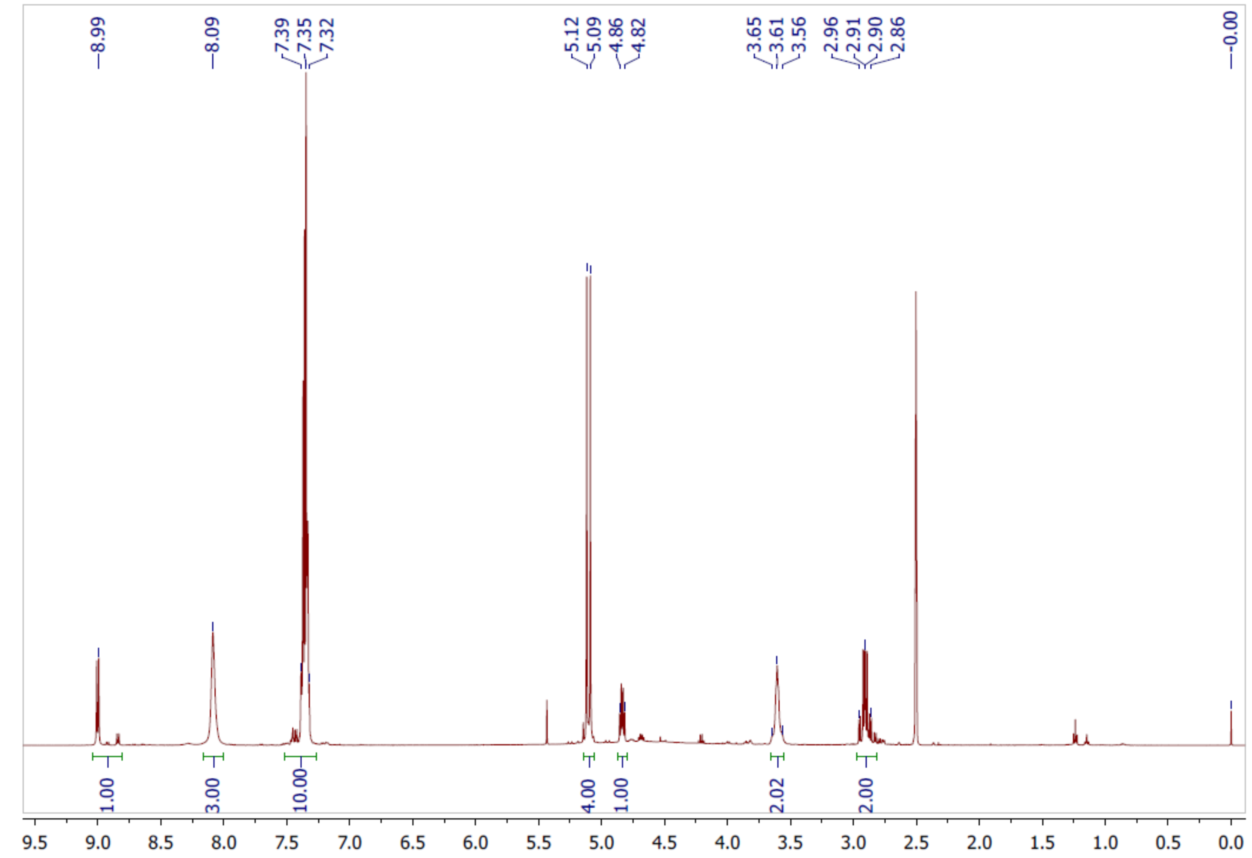

Figure S3. ^{1}H NMR spectrum of compound **5**

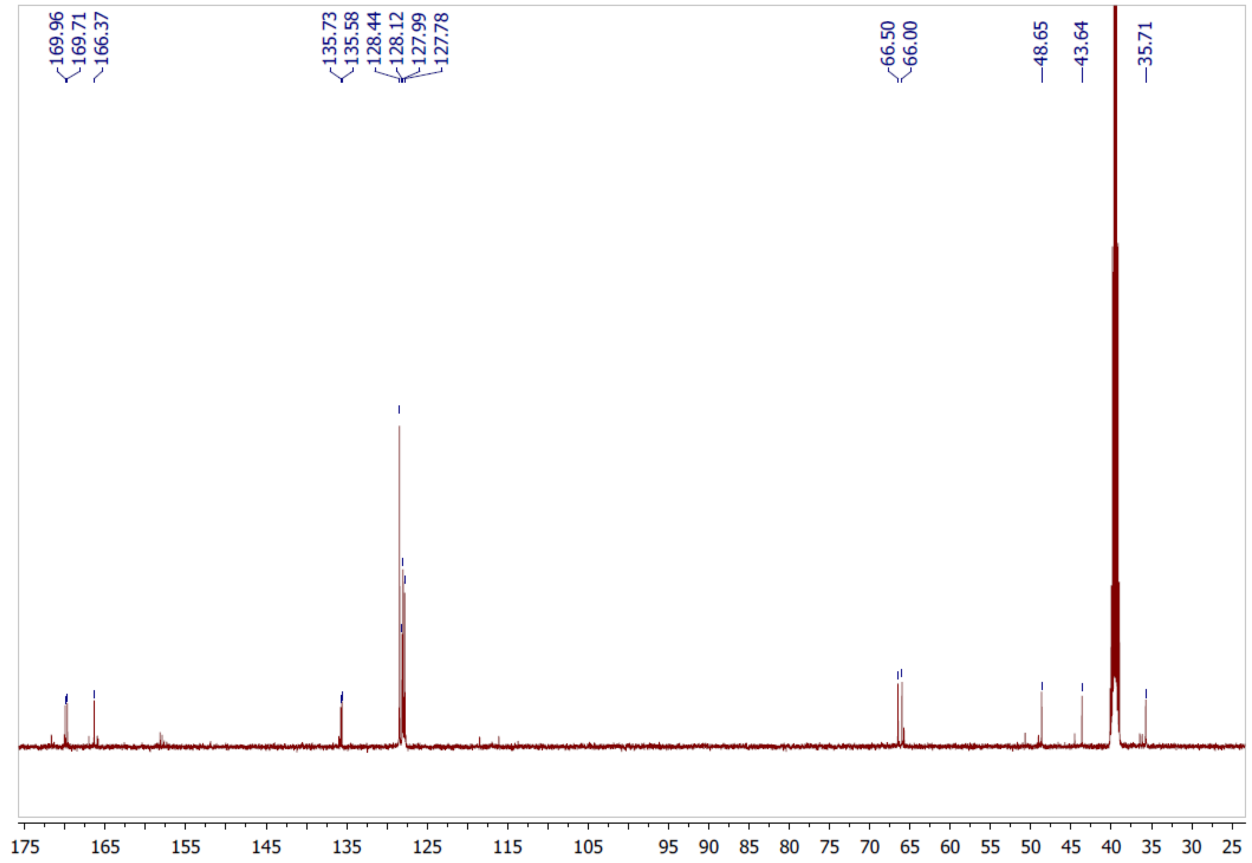

Figure S4. ^{13}C NMR spectrum of compound **5**

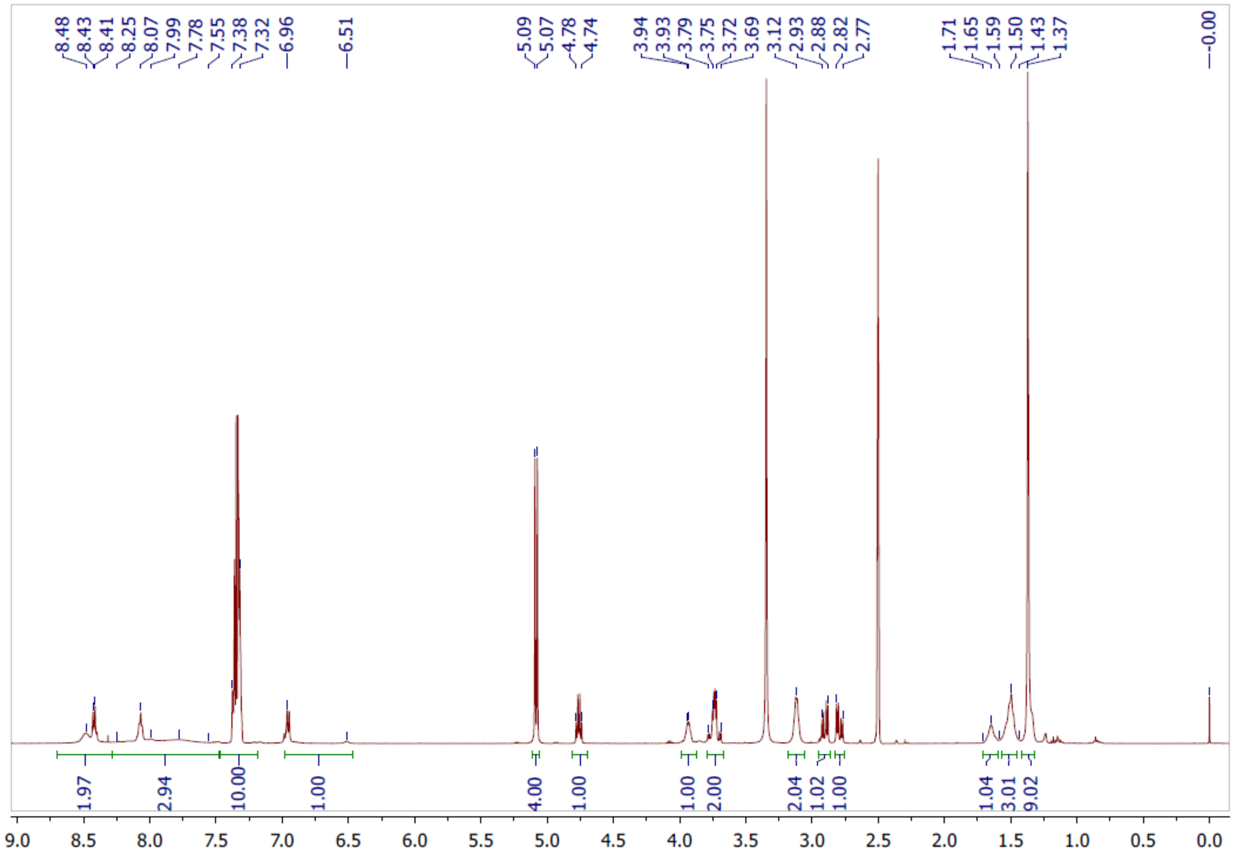

Figure S5. ^{1}H NMR spectrum of compound **6**

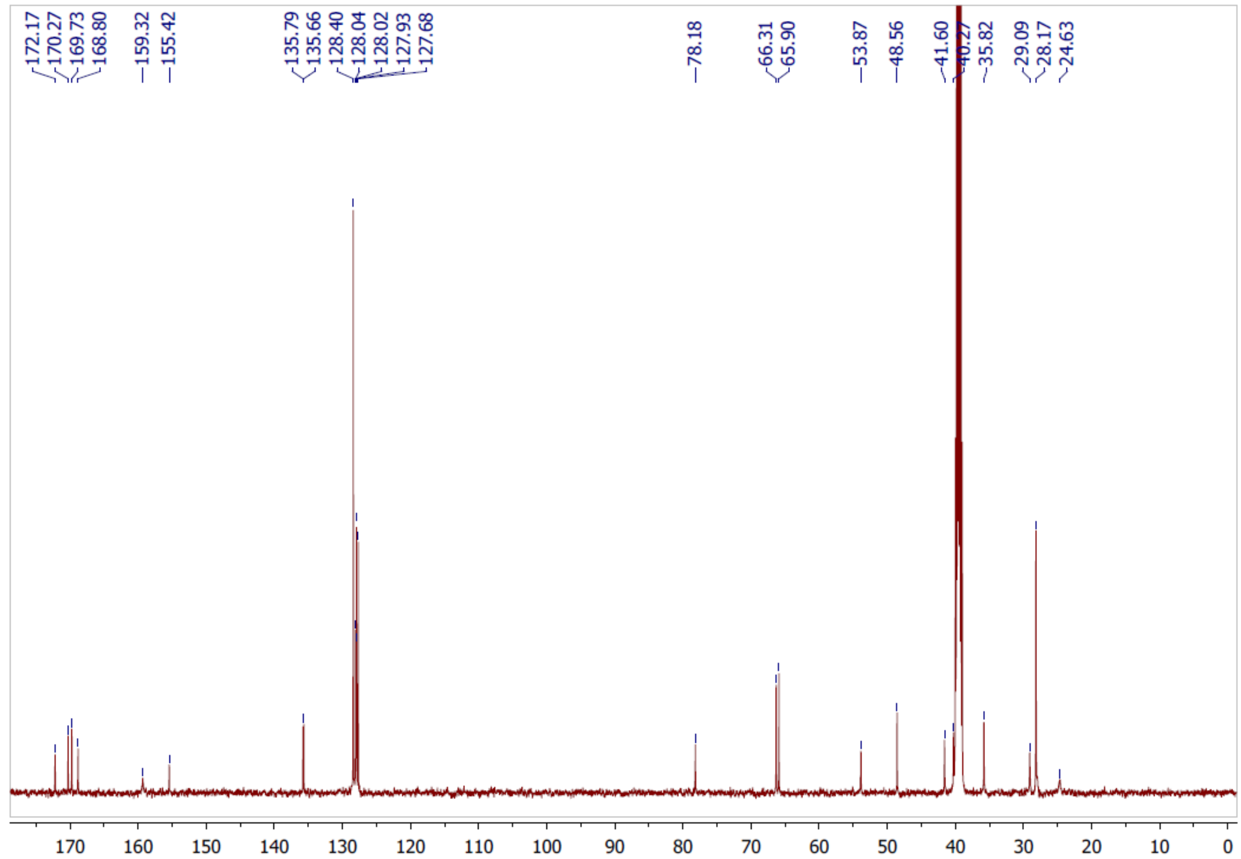

Figure S6. ^{13}C NMR spectrum of compound **6**

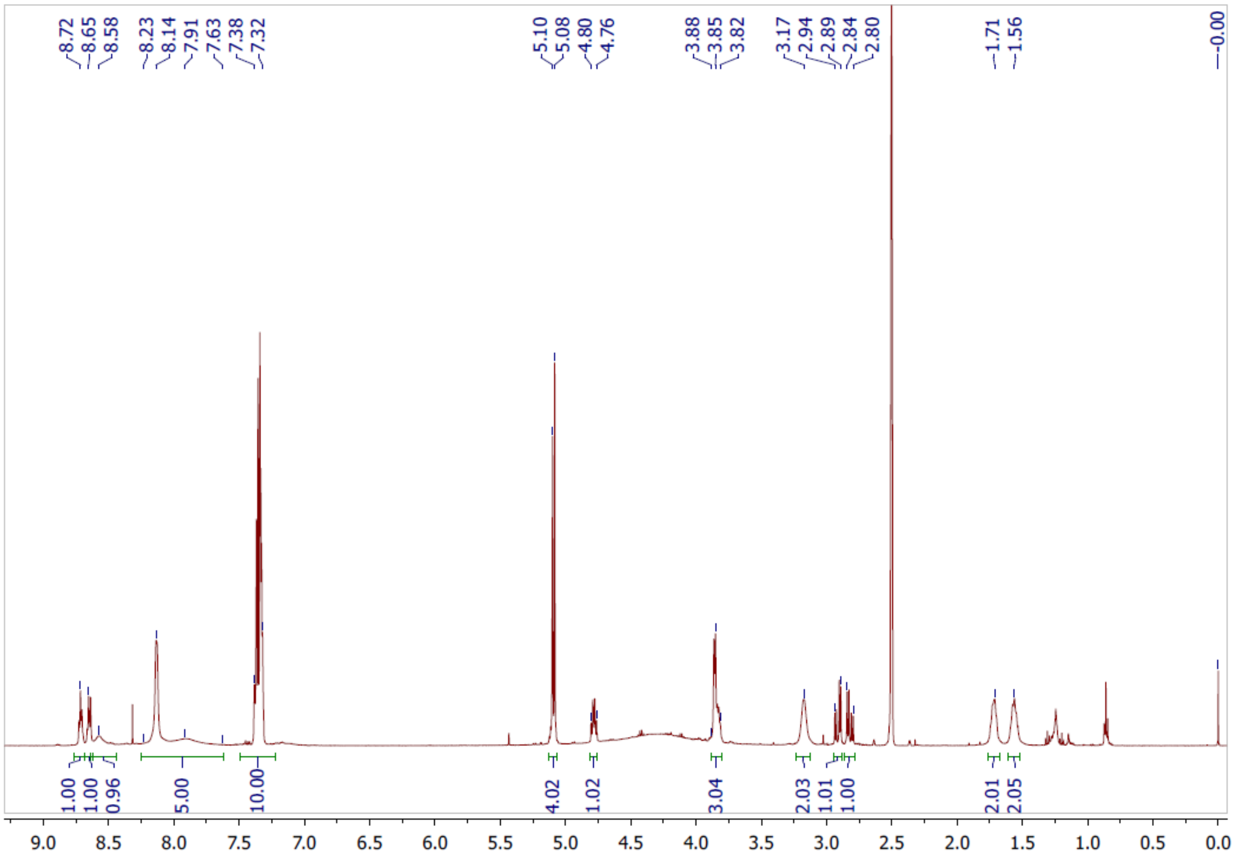

Figure S7. ^{1}H NMR spectrum of compound **7**

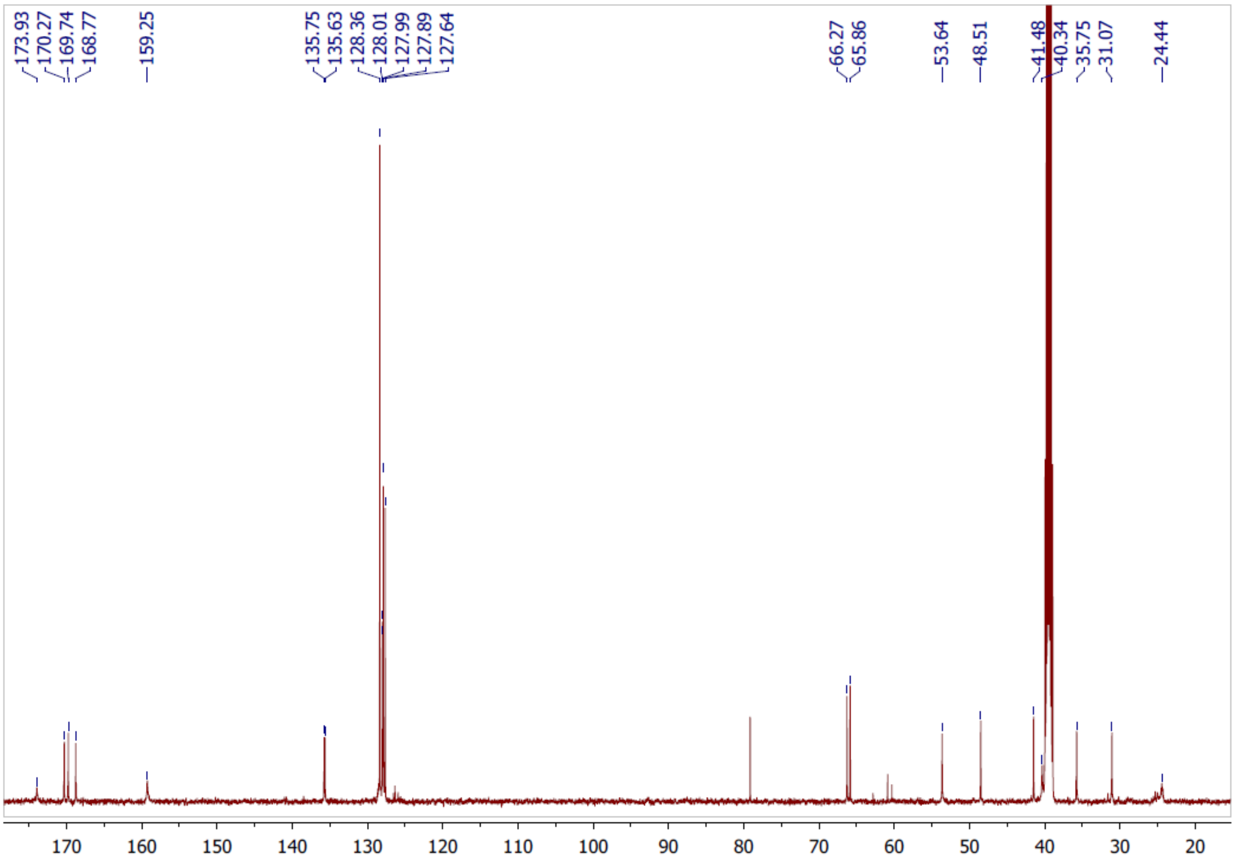

Figure S8. ^{13}C NMR spectrum of compound **7**

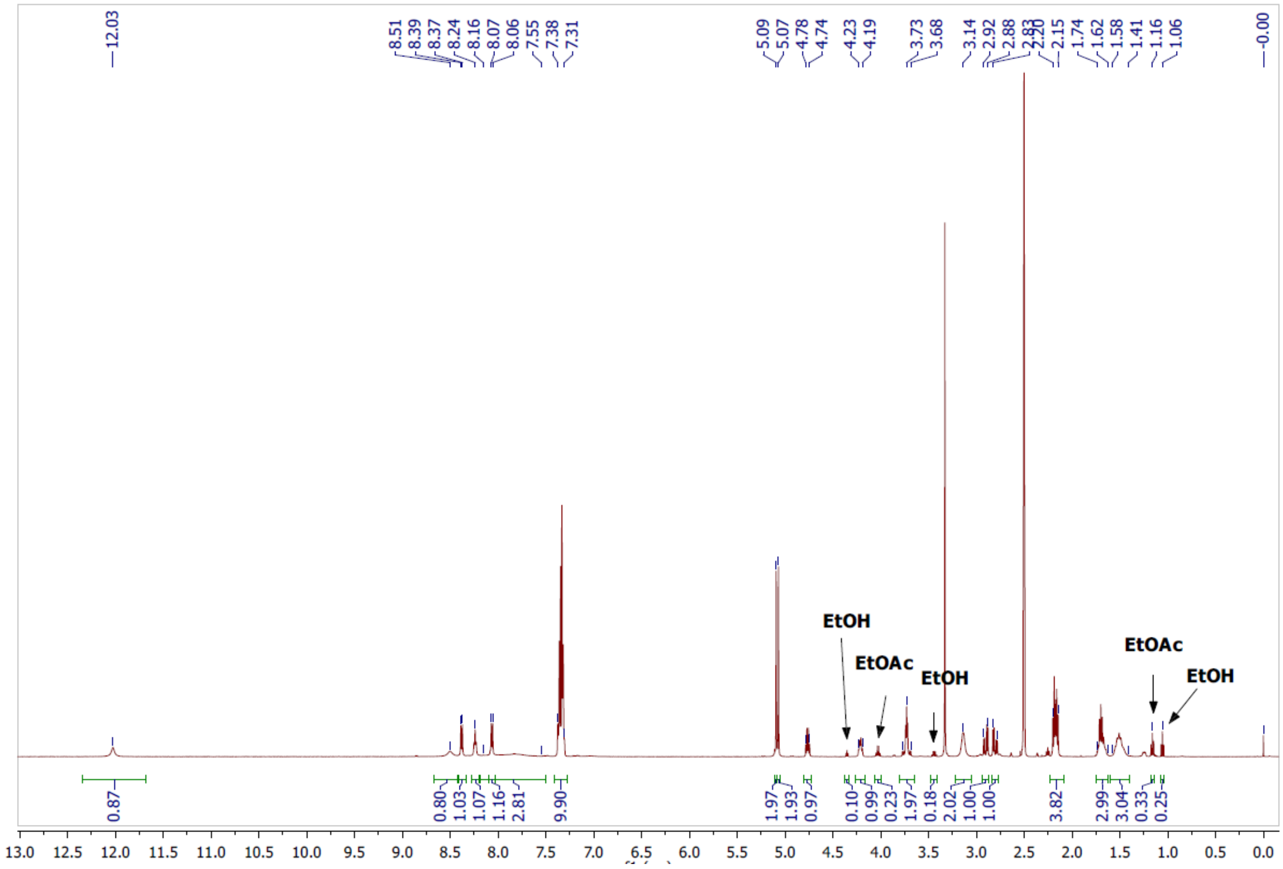

Figure S9. ^{1}H NMR spectrum of compound **2c**

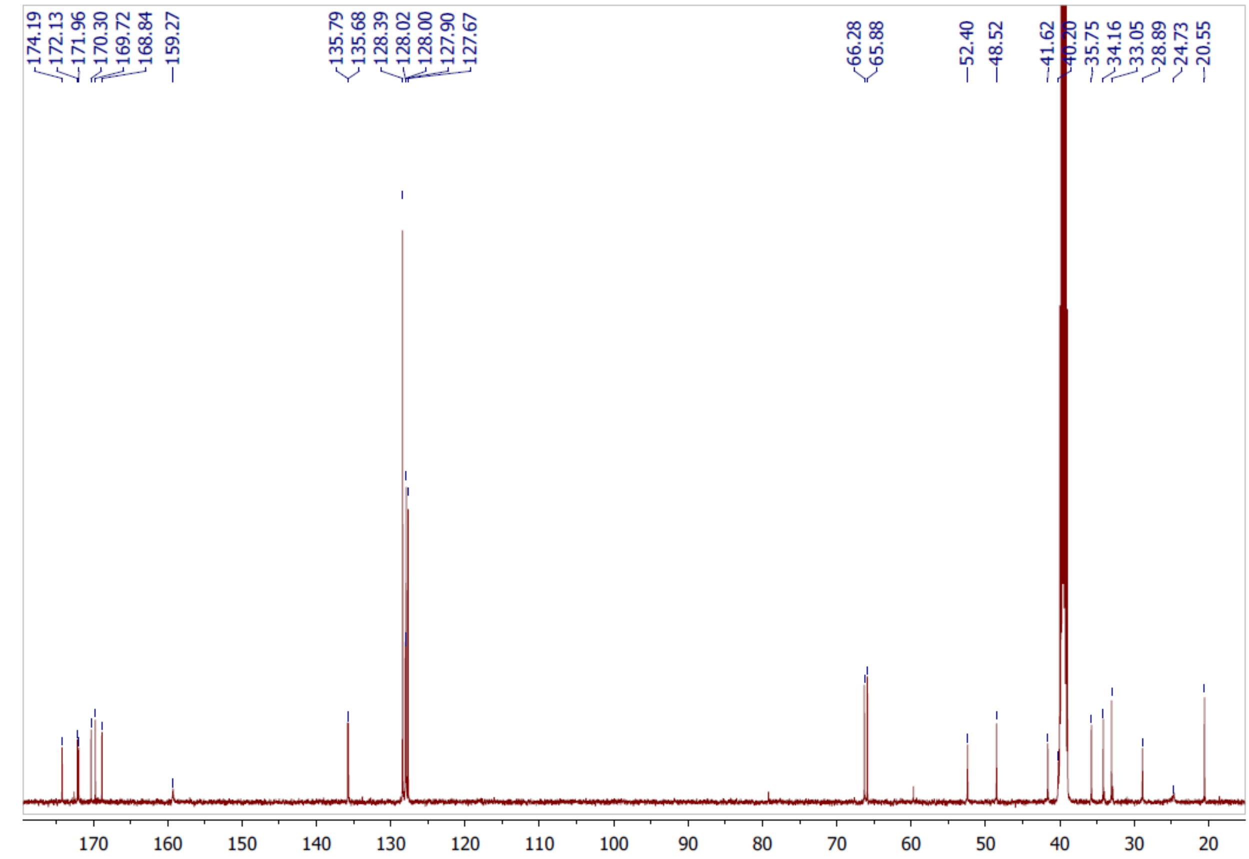

Figure S10. ^{13}C NMR spectrum of compound **2c**

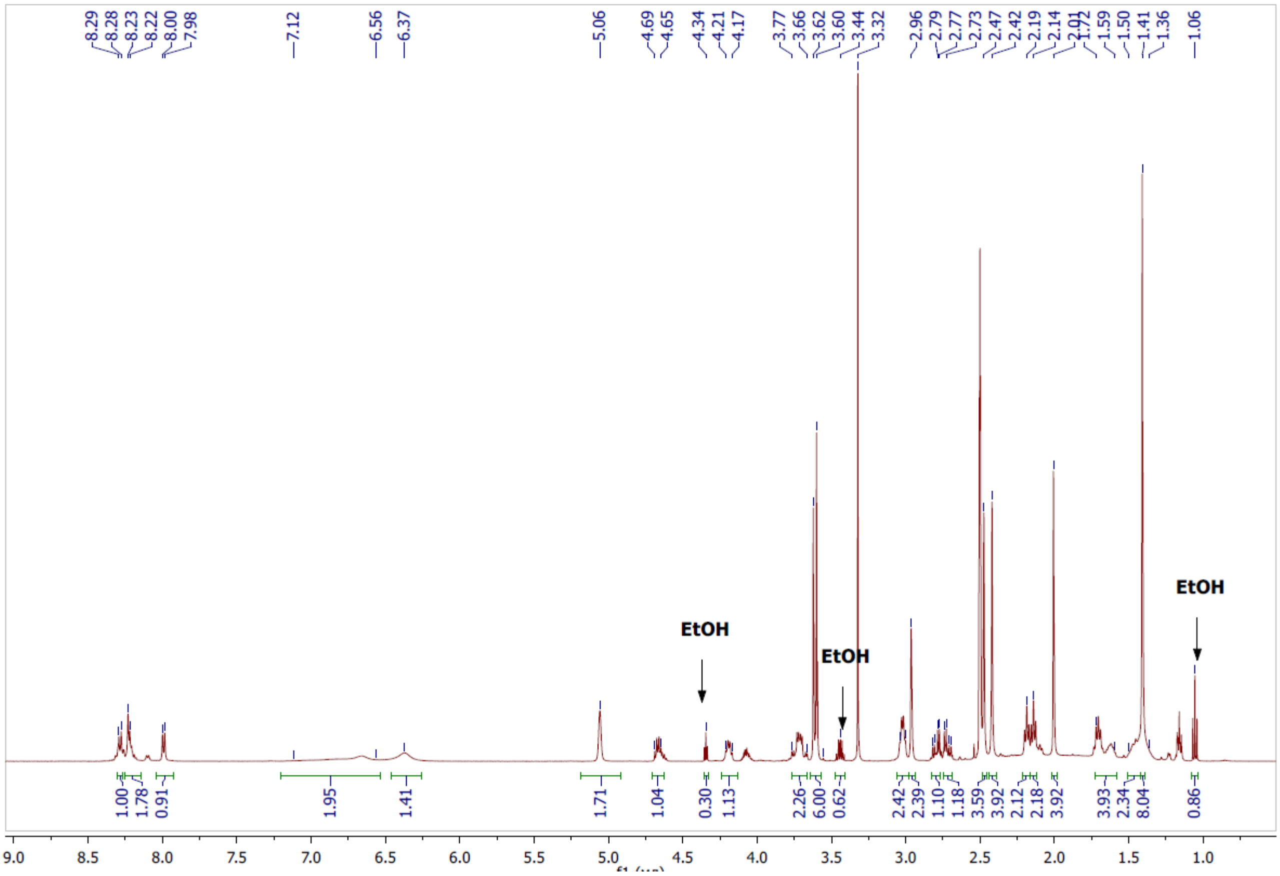

Figure S11. ^{1}H NMR spectrum of compound **1a**

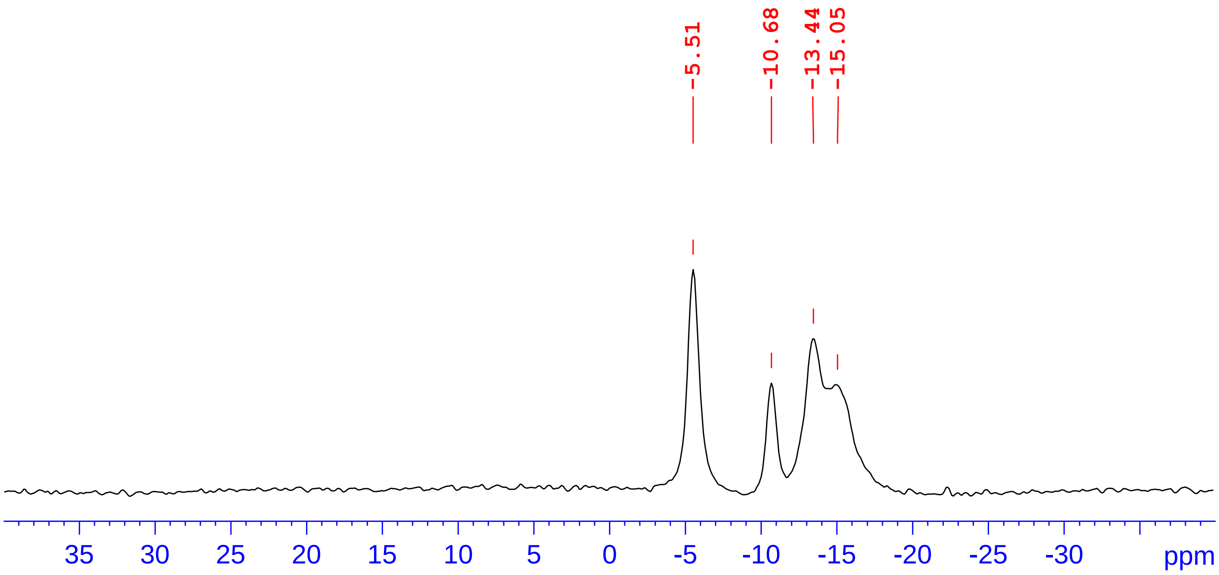

Figure S12. ^{11}B NMR spectrum of compound **1a**

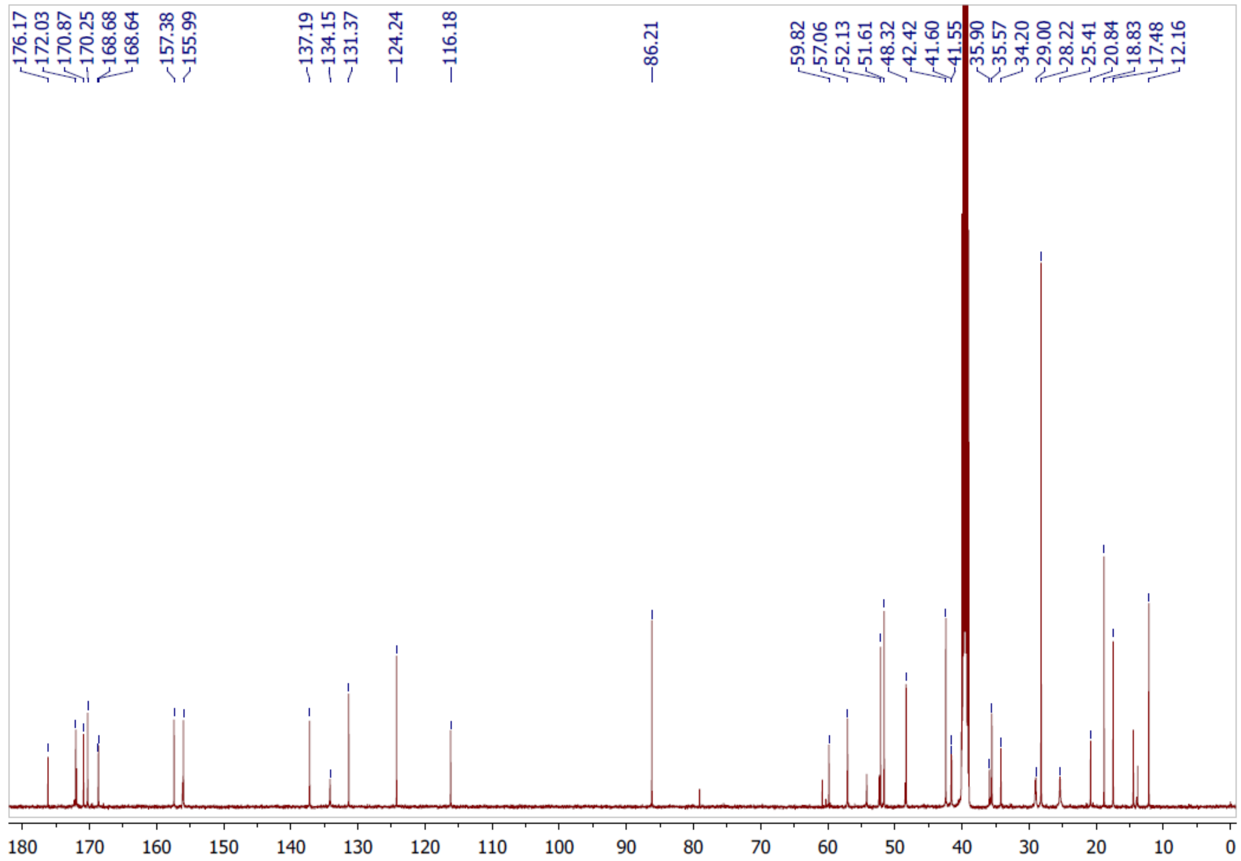

Figure S13. ^{13}C NMR spectrum of compound **1a**

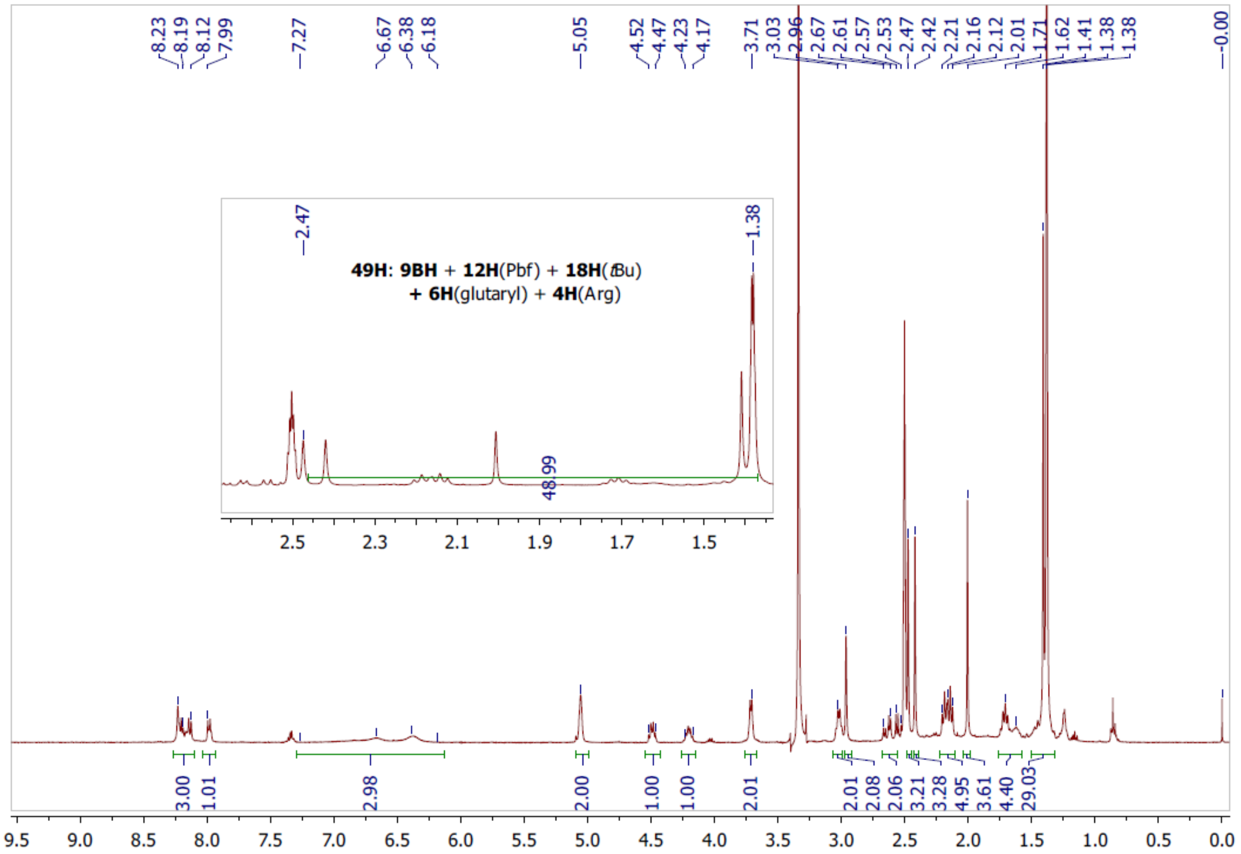

Figure S14. ^{1}H NMR spectrum of compound **1b**

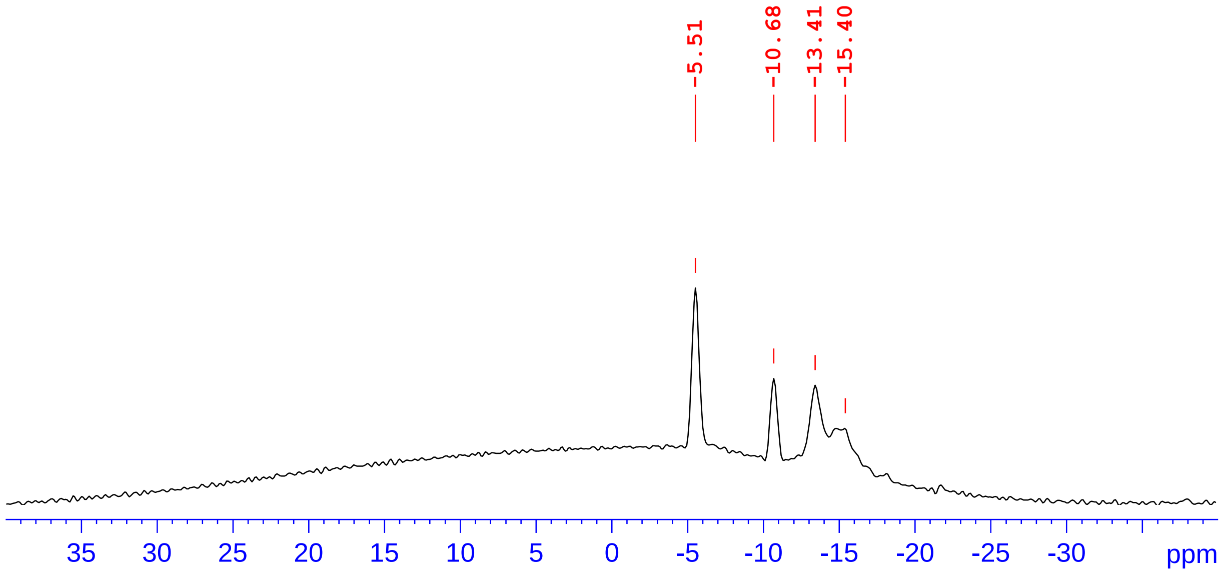

Figure S15. ^{11}B NMR spectrum of compound **1b**

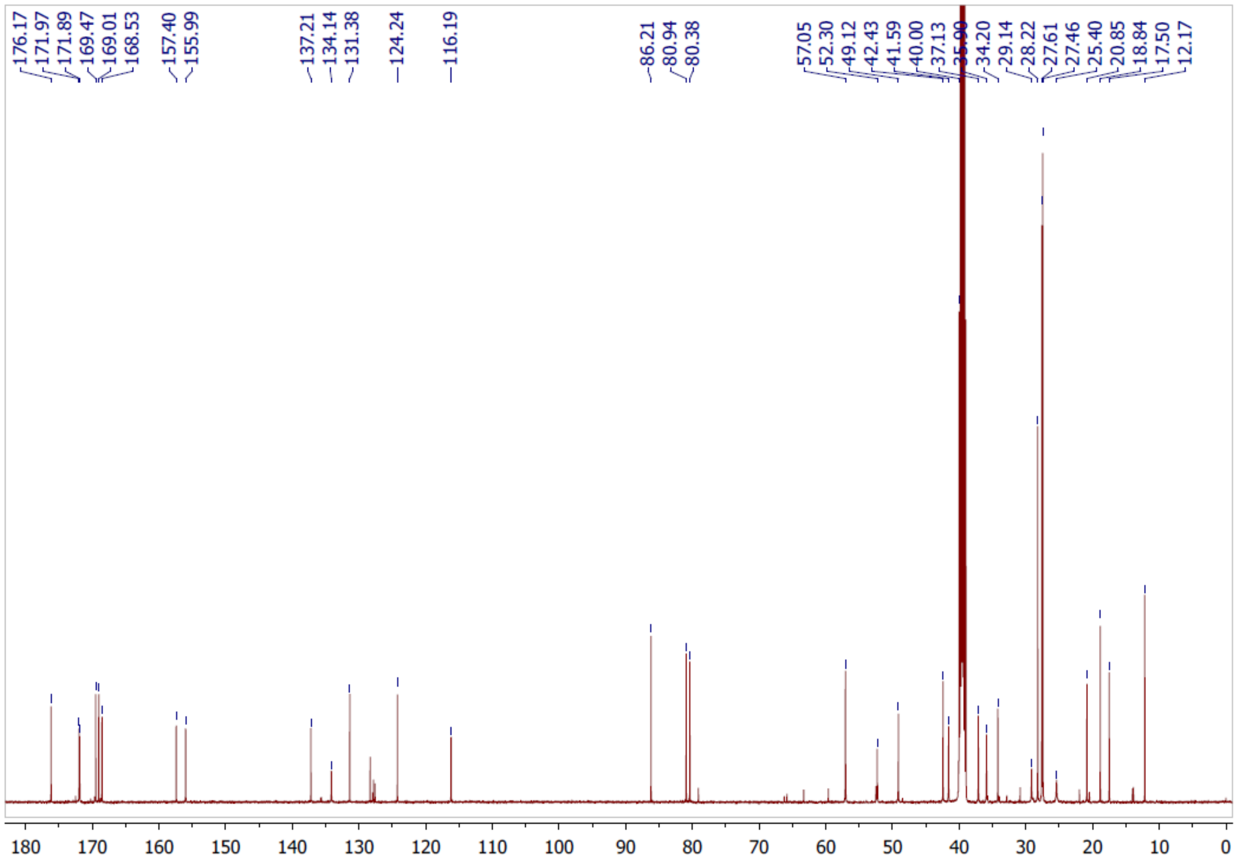

Figure S16. ^{13}C NMR spectrum of compound **1b**

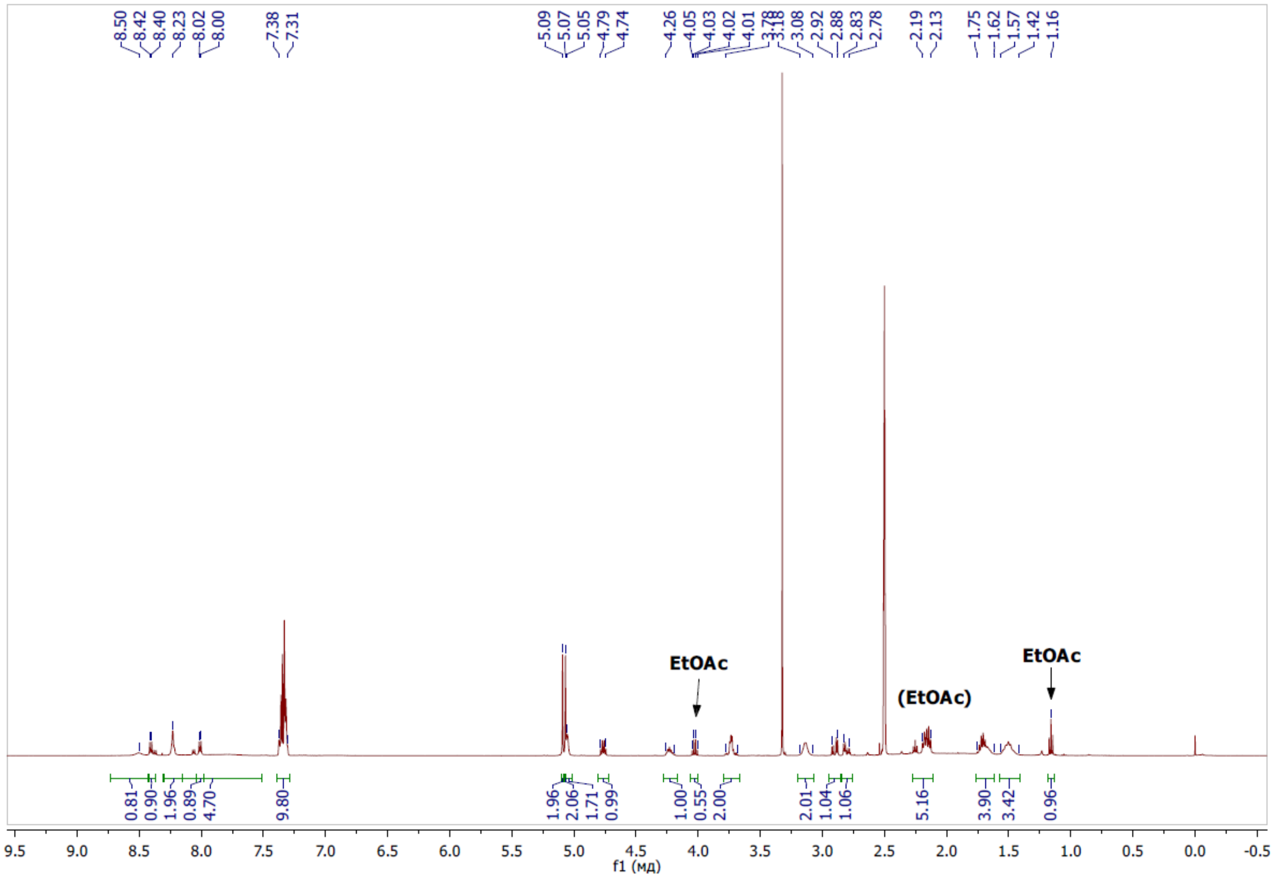

Figure S17. ^{1}H NMR spectrum of compound **1c**

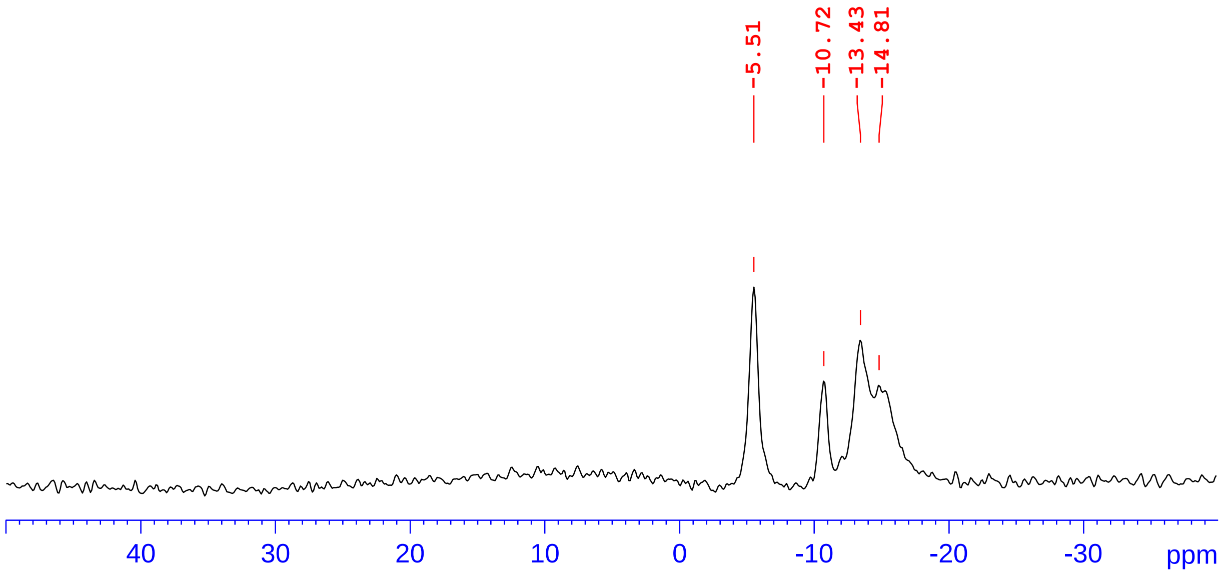

Figure S18. ^{11}B NMR spectrum of compound **1c**

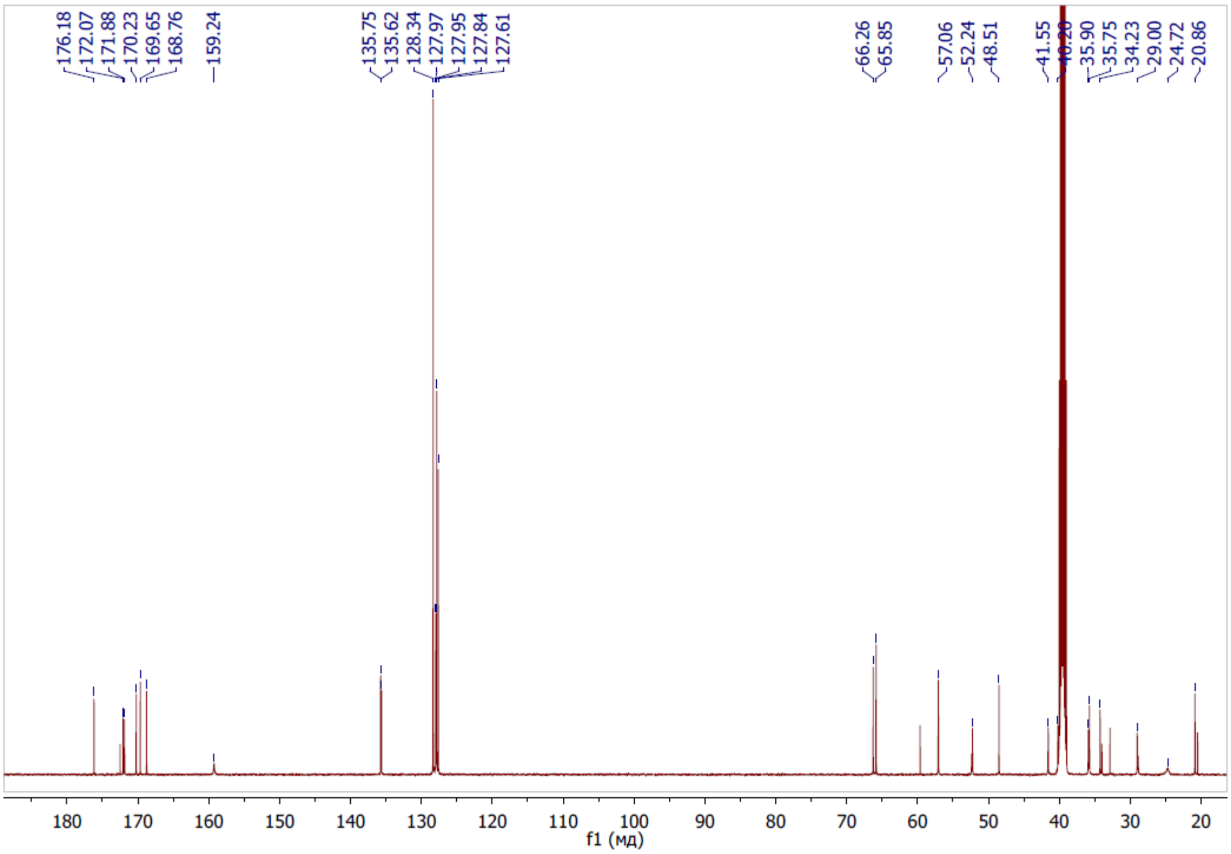

Figure S19. ^{13}C NMR spectrum of compound **1c**

HPLC Data

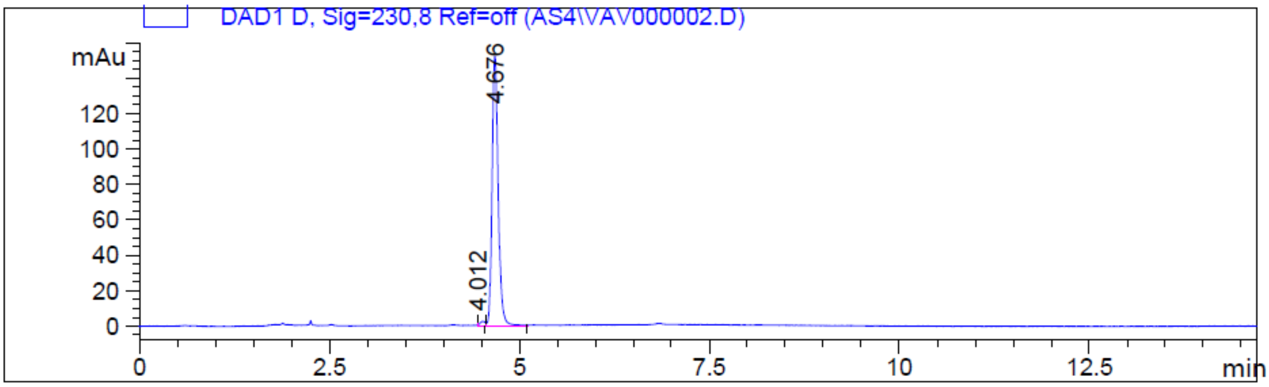

Signal 1: DAD1 D, Sig=230,8 Ref=off

Peak #	RetTime [min]	Type	Width [min]	Area [mAU*s]	Height [mAU]	Area %
1	4.676	VB	0.0870	846.00684	151.79439	98.9607
2	4.012	VB	0.0183	8.88488	1.55413	1.0393
Totals :				854.89172	153.34852	

Figure S20. HPLC of compound **4** (Kromasil 100-5 C18, MeCN–H_2O 1 : 1, 0.8 mL/min, 35 °C, 230 nm)

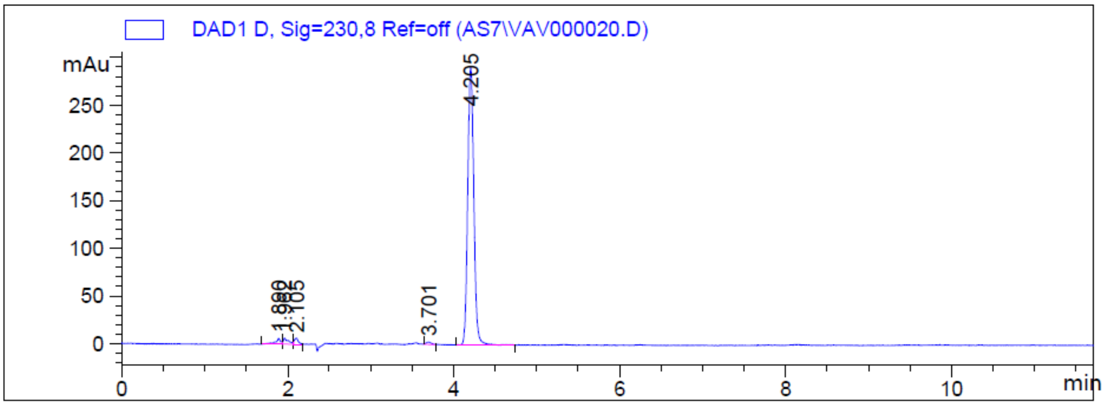

Signal 1: DAD1 D, Sig=230,8 Ref=off

Peak #	RetTime [min]	Type	Width [min]	Area [mAU*s]	Height [mAU]	Area %
1	1.890	MF	0.0719	4.41712	2.14745	0.2807
2	1.962	FM	0.0724	4.57223	2.31812	0.2906
3	2.105	FM	0.0542	3.44363	2.35420	0.2189
4	3.701	MM	0.0744	1.64553	1.21082	0.1046
5	4.205	BB	0.0797	1488.84937	291.11545	99.1052
Totals :				1502.92788	299.14604	

Figure S21. HPLC of compound **6** (Kromasil 100-5 C18, MeCN–H_2O–AcOH 80: 20 : 0.0025, 0.8 mL/min, 35 °C, 230 nm)

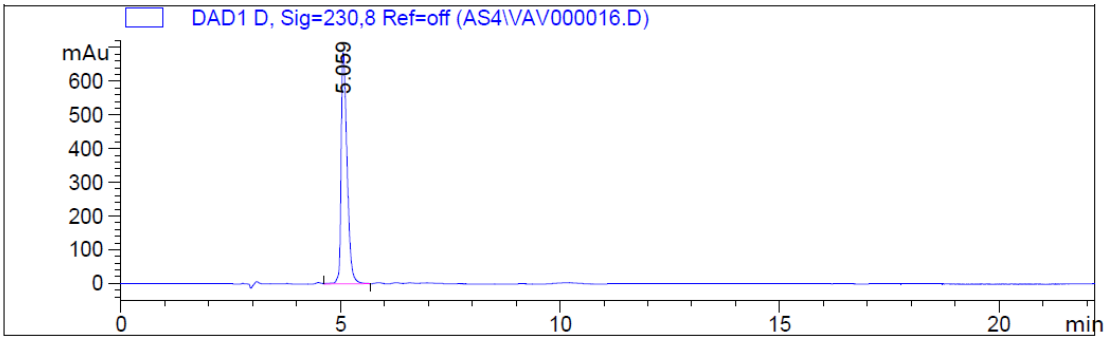


```
Signal 3: DAD1 D, Sig=230,8 Ref=off

Peak RetTime Type  Width     Area      Height     Area
  #   [min]        [min]   [mAU*s]     [mAU]       %
----|-------|----|-------|----------|----------|--------|
   1   5.059 VV    0.1459 6572.58545  685.54456 100.0000

Totals :                  6572.58545  685.54456
```

Figure S22. HPLC of compound **7** (Kromasil 100-5 C18, MeCN–H_2O–CF_3CO_2H 70: 30 : 0.01, 0.8 mL/min, 35 °C, 230 nm)

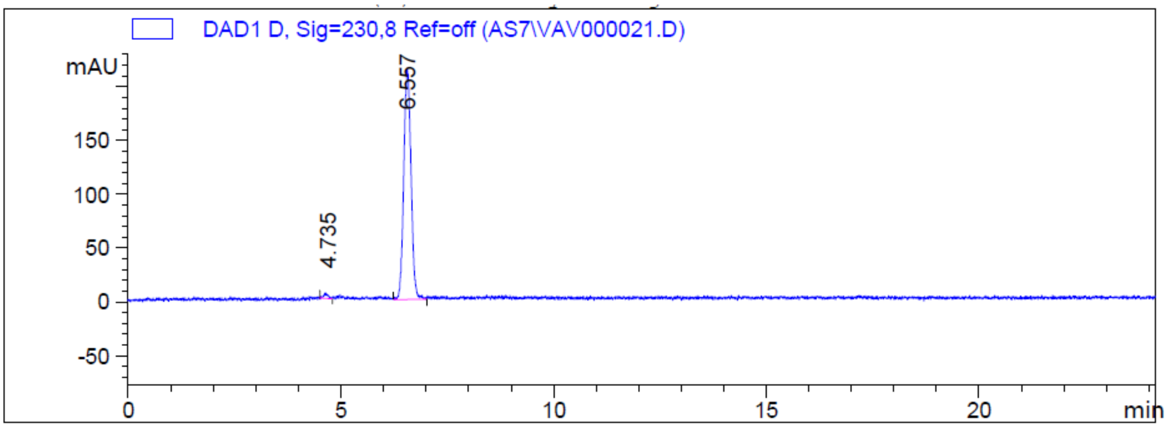


```
Signal 1: DAD1 D, Sig=230,8 Ref=off

Peak RetTime Type  Width     Area      Height     Area
  #   [min]        [min]   [mAU*s]     [mAU]       %
----|-------|----|-------|----------|----------|--------|
   1   4.735 BB    0.0710    7.65632    8.95126   0.2990
   2   6.557 BB    0.1873 2552.85986  213.81601  99.7010

Totals :                  2560.51618  222.76727
```

Figure S23. HPLC of compound **2c** (Kromasil 100-5 C18, MeCN–H_2O–AcOH 60 : 40 : 0.005, 0.8 mL/min, 35 °C, 230 nm)

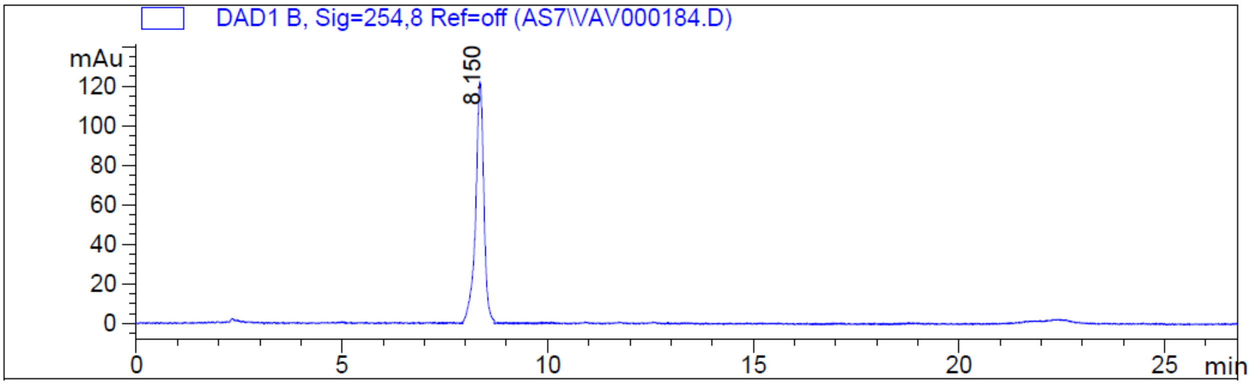

Signal 1: DAD1 B, Sig=254,8 Ref=off

Peak #	RetTime [min]	Type	Width [min]	Area [mAU*s]	Height [mAU]	Area %
1	8.150	VB	0.2490	2318.23071	131.92964	100.0000

Totals : 2318.23071 131.92964

Figure S24. HPLC of compound **1a** (Kromasil 100-5 C18, MeCN–H_2O 1 : 1, 0.8 mL/min, 35 °C, 254 nm)

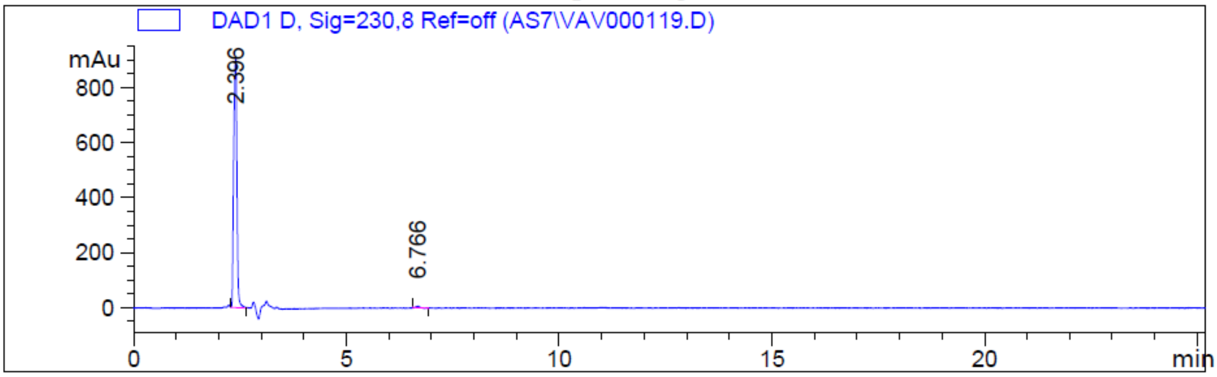

Signal 1: DAD1 D, Sig=230,8 Ref=off

Peak #	RetTime [min]	Type	Width [min]	Area [mAU*s]	Height [mAU]	Area %
1	2.396	BB	0.0862	4706.64990	910.28558	99.7007
2	6.766	BB	0.0644	14.12896	2.73546	0.2993

Totals : 4720.77886 913.02104

Figure S25. HPLC of compound **1b** (Kromasil 100-5 C18, MeCN–H_2O–AcOH 40 : 60 : 0.0025, 0.8 mL/min, 35 °C, 230 nm)

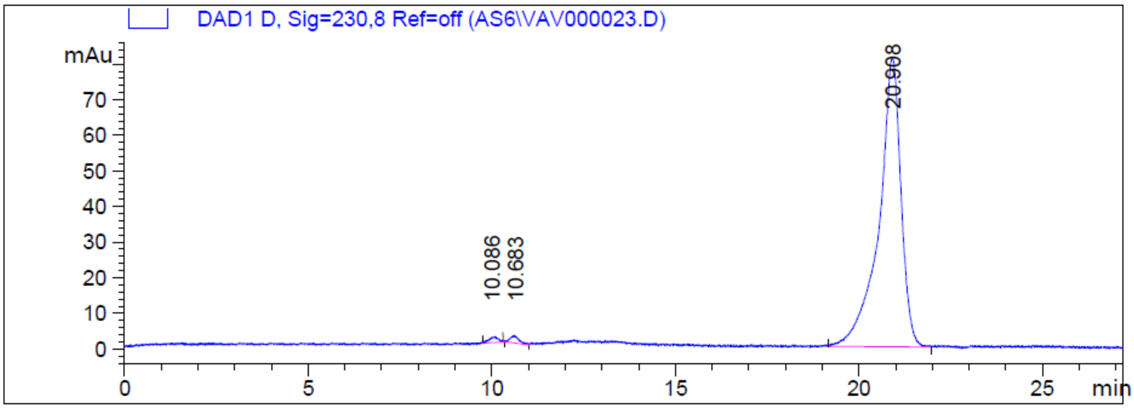

Signal 2: DAD1 D, Sig=230,8 Ref=off

Peak #	RetTime [min]	Type	Width [min]	Area [mAU*s]	Height [mAU]	Area %
1	10.086	BV	0.1274	16.88312	4.01685	0.4941
1	10.683	BV	0.1486	23.63285	4.56981	0.6917
1	20.908	BB	0.5838	3376.25488	81.08988	98.8142

Totals : 3416.77085 89.67654

Figure S26. HPLC of compound **1c** (Kromasil 100-5 C18, MeCN–0.06 M phosphate buffer solution (pH 7.0) 8 : 2, 0.8 mL/min, 35 °C, 230 nm)
